# Direct IVF versus prior laparoscopic cystectomy for ovarian endometrioma ≥4 cm: cumulative live birth rate and implications for fertility preservation — a retrospective cohort study

**DOI:** 10.3389/fendo.2026.1851907

**Published:** 2026-07-31

**Authors:** Yan Feng, Xiaoyu Yang, Huanhuan Chen, Lei Zhang, Chenchen Cui, Lu Wang, Qiaohua He, Linlin Liang

**Affiliations:** 1 Department of Gynecology, Henan International Joint Laboratory of Early Diagnosis and Treatment of Gynecological Malignant Tumors, Henan Provincial People’s Hospital, Zhengzhou, Henan, China; 2 Zhengzhou University People’s Hospital, Zhengzhou, Henan, China; 3 Reproductive Medicine Center, Henan Provincial People’s Hospital, People’s Hospital of Zhengzhou University, Zhengzhou, Henan, China; 4 Henan Joint International Research Laboratory of Reproductive Bioengineering, Zhengzhou, Henan, China

**Keywords:** cumulative live birth rate, fertility preservation, *in vitro* fertilization, laparoscopic cystectomy, ovarian endometrioma, ovarian reserve

## Abstract

**Background:**

Current guidelines recommend laparoscopic cystectomy for ovarian endometrioma (OMA) ≥4 cm before fertility treatment, yet cystectomy inevitably damages ovarian reserve. While recent large-scale studies have confirmed that cystectomy reduces antimüllerian hormone (AMH) levels and oocyte yield, whether this translates into impaired cumulative live birth rate (CLBR)—the most clinically relevant IVF outcome—remains inadequately addressed.

**Methods:**

This retrospective cohort study included 394 infertile patients with OMA undergoing their first IVF cycle at a single tertiary center (January 2016–December 2022). Patients were stratified into Group A (non-surgical OMA <4 cm, n=116), Group B (non-surgical OMA ≥4 cm, n=47), and Group C (surgical OMA ≥4 cm, n=231). The primary outcome was CLBR. Multivariable logistic regression (primary analysis of CLBR), propensity score matching (PSM), and inverse probability of treatment weighting (IPTW) were employed. Multiple linear regression was used as an exploratory analysis of oocyte yield.

**Results:**

In non-surgically managed patients, OMA size did not independently predict CLBR (Group B vs. A: OR = 1.06, P = 0.882). Among patients with OMA ≥4 cm, prior cystectomy was associated with significantly lower AMH (1.98 vs. 3.16 ng/mL, P = 0.001), fewer retrieved oocytes (median 7 vs. 11, P = 0.001), and impaired embryological outcomes. However, CLBR did not differ significantly between surgical and non-surgical management (52.4% vs. 59.6%, P = 0.370; adjusted OR = 0.77, 95% CI: 0.38–1.56, P = 0.469). PSM (OR = 0.67, P = 0.374) and IPTW (OR = 0.77, P = 0.448) confirmed these findings. Patient age (OR = 0.92, P = 0.030) and number of transferred embryos (OR = 2.21, P = 0.014) were the only independent predictors of CLBR.

**Conclusions:**

Pre-IVF laparoscopic cystectomy for OMA ≥4 cm significantly compromises ovarian reserve and IVF efficiency and this study did not detect a significant improvement in CLBR, although the comparison was underpowered to exclude clinically meaningful differences. Direct IVF may warrant consideration as an alternative strategy for patients without absolute surgical indications. When surgery is planned, preoperative fertility preservation should be considered. These findings require validation in prospective randomized trials.

## 1 Introduction

The clinical management of infertile patients with ovarian endometrioma (OMA) ≥4 cm presents a significant therapeutic dilemma. Current guidelines recommend laparoscopic cystectomy before fertility treatment for OMA measuring ≥4 cm in diameter (1, 2). However, this recommendation creates a fundamental tension: while surgery aims to optimize the pelvic environment for conception, it inevitably removes healthy ovarian tissue along with the cyst wall, potentially compromising the very ovarian reserve upon which successful IVF depends. As a result, reproductive endocrinologists increasingly question whether pre-IVF cystectomy truly improves pregnancy outcomes or whether direct IVF might achieve comparable results while preserving ovarian function. This unresolved controversy has profound implications for patient counseling and treatment sequencing, yet remains inadequately addressed by current evidence.

Endometriosis affects approximately 10% of reproductive-age women, with infertility reported in 30–50% of affected individuals (3–5). OMA, the most prevalent form of ovarian endometriosis, adversely affects fertility through multiple pathways, including local inflammation, pelvic adhesions, hormonal dysregulation, impaired folliculogenesis, and reduced oocyte quality (6, 7). In the context of assisted reproduction, women with OMA typically require higher gonadotropin doses, exhibit diminished ovarian responsiveness, and demonstrate lower pregnancy rates compared with non-endometriosis controls (8, 9).

Laparoscopic cystectomy has been advocated for OMA management on the grounds that it may alleviate symptoms, reduce the risk of rupture, and improve the pelvic environment (10) (11). However, surgery represents a double-edged sword. Multiple studies have demonstrated that cystectomy significantly compromises ovarian reserve: following unilateral cystectomy, the operated ovary shows markedly reduced antral follicle count (AFC) and oocyte yield compared with the contralateral side (Daniilidis et al., 2023; Shandley et al., 2023), while bilateral cystectomy is associated with 30–50% reductions in antimüllerian hormone (AMH) levels and substantially fewer retrieved oocytes (12, 13) (14), a finding corroborated by a meta-analysis demonstrating significant postoperative AMH decline following both unilateral and bilateral excision (15). This surgical damage to ovarian reserve is largely irreversible, attributable to thermal injury from electrocautery hemostasis, inadvertent excision of functional cortex, and disruption of ovarian vascularity. Paradoxically, while surgery aims to improve fertility, it may inadvertently reduce the reproductive potential upon which IVF success critically depends. This concern is particularly salient given that patient age-closely linked to ovarian reserve-remains the most important predictor of IVF outcomes, and that delays incurred by surgical intervention may themselves compromise reproductive success.

Despite the clinical importance of this question, direct evidence comparing surgical versus non-surgical management using cumulative live birth rate (CLBR)-the most clinically relevant IVF outcome-remains limited. Most existing studies have focused on surrogate endpoints such as oocyte yield, fertilization rates, or clinical pregnancy per transfer, which do not capture the ultimate goal of fertility treatment. A landmark randomized controlled trial by Garcia-Velasco et al. (2004) demonstrated no significant difference in pregnancy rates between surgical and expectant management before IVF (16), and a meta-analysis by Hamdan et al. (2015) confirmed that the presence of OMA was associated with reduced oocyte yield but did not significantly impair clinical pregnancy rates (17). A subsequent systematic review similarly reported comparable IVF results between women with untreated OMA and those who underwent pre-IVF surgery (18). However, robust comparisons specifically examining CLBR in patients with OMA ≥ 4 cm remain scarce, as most existing studies have employed per-cycle or per-transfer pregnancy rates rather than cumulative outcomes encompassing all fresh and frozen-thawed embryo transfers from a single stimulation cycle. Furthermore, it remains unclear whether patients with conservatively managed OMA ≥ 4 cm experience inferior outcomes compared with those with smaller cysts-information essential for counseling patients who decline or are not candidates for surgery. This limitation persists in recent large-scale investigations. Li et al. (2026), in a retrospective cohort of 833 patients published in Frontiers in Endocrinology, confirmed that prior endometriotic cystectomy significantly reduces AMH, AFC, and oocyte yield compared with both non-surgical endometriosis patients and non-endometriosis controls (19). However, their study did not include pregnancy or live birth data, leaving the most clinically relevant question—whether surgical damage to ovarian reserve translates into impaired cumulative reproductive outcomes—unanswered.

The 2022 Chinese Expert Consensus on Fertility Preservation in Endometriosis Patients has highlighted an important and increasingly recognized clinical consideration: when surgical intervention is planned, fertility preservation (oocyte or embryo cryopreservation) should be considered prior to cystectomy, particularly in patients with already diminished ovarian reserve (20). This recommendation acknowledges that even patients with apparently normal preoperative ovarian reserve may experience significant postoperative decline. Nonetheless, the extent to which pre-IVF surgery-as opposed to direct IVF-affects ultimate reproductive outcomes remains the critical unanswered question that should inform this decision.

The present study was designed to extend the existing evidence by addressing two key clinical questions in infertile patients with OMA that remain unresolved despite recent large-scale investigations focused on surrogate endpoints. First, we examined whether OMA size independently affects IVF outcomes in non-surgically managed patients, thereby informing the decision of whether expectant management is appropriate for OMA ≥4 cm. Second, and more importantly, we compared IVF outcomes between surgical and non-surgical management of OMA ≥4 cm to determine whether pre-IVF cystectomy improves CLBR. Through multivariable regression analysis and propensity score methods, including propensity score matching (PSM) and inverse probability of treatment weighting (IPTW), we aimed to provide evidence-based guidance for individualized treatment sequencing in this challenging patient population.

## 2 Materials and methods

### 2.1 Study population and participants

This was a retrospective cohort study of 394 infertile patients with ovarian endometrioma who attended the Reproductive Medicine Center of Henan Provincial People’s Hospital between January 2016 and December 2022. In the non-surgical group, the diagnosis of OMA was established by transvaginal ultrasound (TVS) performed at our institution, whereas in the surgical group, the diagnosis was histologically confirmed following laparoscopic cystectomy. TVS has demonstrated high diagnostic accuracy for ovarian endometrioma, with reported sensitivity and specificity ranging approximately from 86.5% to 95% and 95% to 99%, respectively (21–23), with characteristic sonographic findings including a well-circumscribed cystic lesion with thickened walls and homogeneous ground-glass echogenicity. TVS is widely recognized as a reliable and non-invasive diagnostic modality for OMA (24).

Inclusion criteria were: (1) age < 40 years; (2) first IVF cycle at our center; (3) normal semen parameters in the male partner.

Exclusion criteria were: (1) recurrent ovarian endometrioma; (2) known genetic disorders or chromosomal abnormalities in either partner; (3) prior oophorectomy or congenital uterine/ovarian anomalies; (4) endocrine disorders, including thyroid dysfunction, diabetes mellitus, or polycystic ovary syndrome (PCOS); (5) uterine pathology, including adenomyosis, adenomyoma, uterine malformations (unicornuate, bicornuate, or septate uterus), or uterine fibroids; (6) preimplantation genetic testing (PGT) cycles; (7) donor oocyte cycles; (8) severe male factor infertility (severe oligozoospermia or azoospermia).

Adenomyosis was specifically excluded because it frequently coexists with endometriosis and is a recognized independent modifier of both implantation efficiency and obstetric outcomes (25, 26). Including patients with concomitant adenomyosis would have introduced an additional confounding variable that could not be adequately controlled for in the present analysis.

### 2.2 Study groups

**Figure 1:**
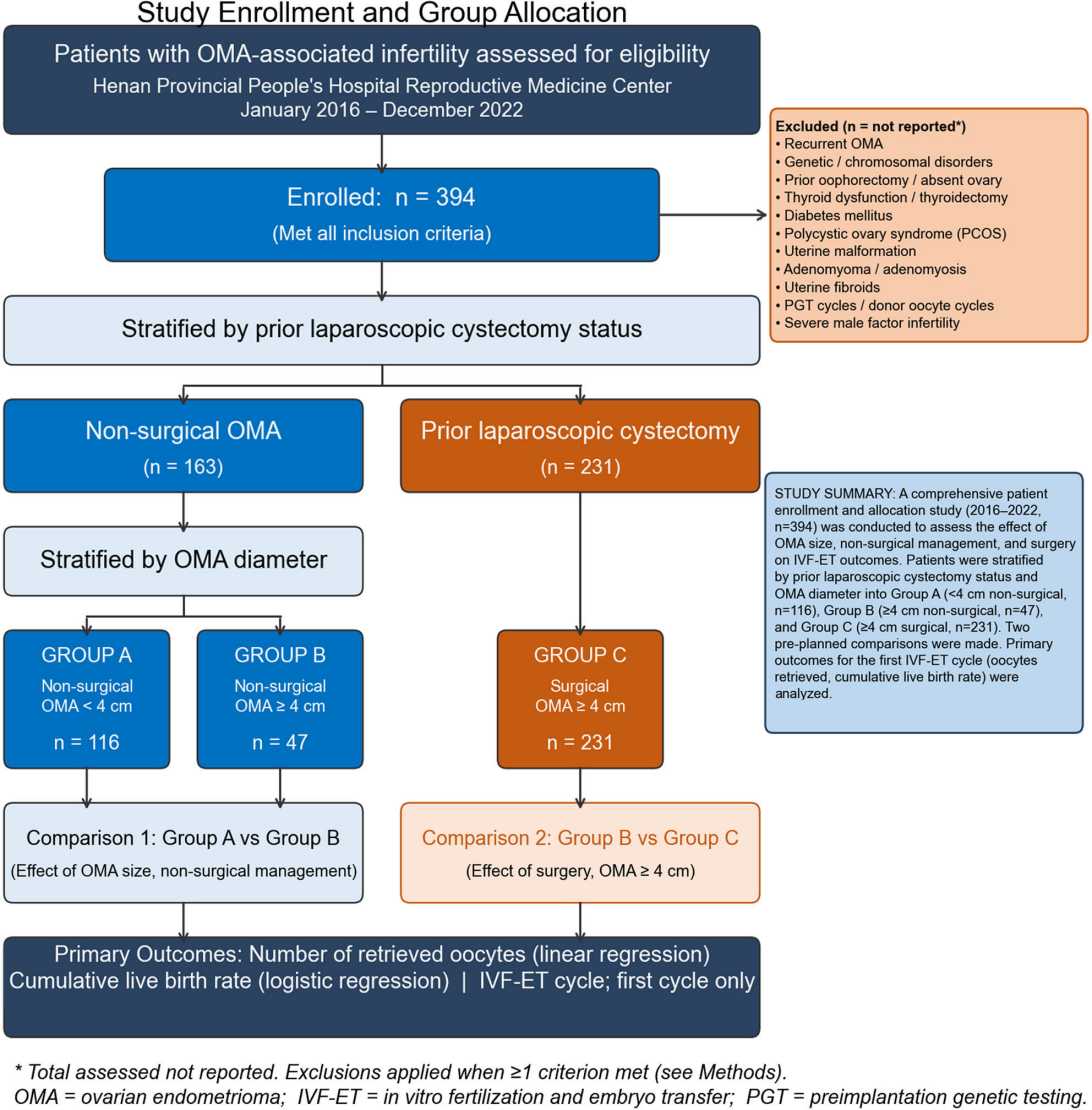
Study enrollment and group allocation flowchart. Infertile patients with ovarian endometrioma (OMA) undergoing their first IVF cycle at Henan Provincial People’s Hospital Reproductive Medicine Center between January 2016 and December 2022 were stratified according to two clinical variables: prior surgical status (laparoscopic cystectomy vs. no surgery) and OMA diameter (<4 cm vs. ≥4 cm). This yielded three mutually exclusive groups: Group A (non-surgical OMA <4 cm, n = 116), Group B (non-surgical OMA ≥4 cm, n = 47), and Group C (surgical OMA ≥4 cm, n = 231). Two primary comparisons were conducted: Group A vs. Group B (effect of OMA size) and Group B vs. Group C (effect of surgical intervention). A study summary box provides a brief synopsis of the design and primary outcomes. OMA, ovarian endometrioma; IVF-ET, *in vitro* fertilization and embryo transfer; PGT, preimplantation genetic testing.

Non-surgical OMA patients were stratified by cyst diameter into two groups: Group A, non-surgical OMA < 4 cm (n = 116); and Group B, non-surgical OMA ≥ 4 cm (n = 47). Patients with OMA ≥ 4 cm were further categorized according to whether they had undergone prior surgical intervention: Group B, non-surgical OMA ≥ 4 cm (n = 47; same as above); and Group C, surgical OMA ≥ 4 cm (n = 231). The patient selection and group allocation process is illustrated in **Figure 1**.

### 2.3 Controlled ovarian stimulation and oocyte retrieval

Ovarian reserve was assessed by measuring serum basal reproductive hormone levels on menstrual cycle days 2–3 using an automated electrochemiluminescence immunoassay system (Cobas e601, Roche Diagnostics, Mannheim, Germany), and by transvaginal ultrasound assessment of the bilateral antral follicle count (AFC) using a high-resolution ultrasound system (Voluson E8, GE Healthcare, Zipf, Austria). COS protocols were individualized by the attending physicians based on patient age and ovarian reserve parameters. Follicular growth and endometrial development were monitored by serial TVS, with concurrent measurement of serum FSH, estradiol (E2), luteinizing hormone (LH), and progesterone (P) to guide gonadotropin (Gn) (Gonal-f, Merck Serono, Darmstadt, Germany) dose adjustments. Final oocyte maturation was triggered when at least two follicles reached ≥18 mm or three follicles reached ≥17 mm in mean diameter, with consideration of serum LH, E2, and P levels on the trigger day. Trigger agents included human chorionic gonadotropin (hCG) (Ovidrel, Merck Serono, Darmstadt, Germany, or urinary hCG, Livzon Pharmaceutical Group, Shenzhen, China), a gonadotropin-releasing hormone agonist (GnRH-a) (Merck Serono, Darmstadt, Germany), or a dual trigger (hCG + GnRH-a). Transvaginal ultrasound-guided oocyte retrieval was performed 34–36 hours later using a single-lumen aspiration needle (Vitrolife, Gothenburg, Sweden).

### 2.4 *In vitro* fertilization and embryo culture

Following oocyte retrieval, insemination was performed by conventional IVF or intracytoplasmic sperm injection (ICSI) according to male partner semen quality. Fertilization was assessed 16–18 hours post-insemination by evaluating pronuclear and polar body status. On day 3 post-insemination, embryos were graded using a five-tier scoring system (Grades I–V); Grades I and II were designated as top-quality embryos. For extended culture, culture medium(G-Series sequential media, Vitrolife, Gothenburg, Sweden) was replaced on day 3, with blastocyst development assessed on days 5 and 6. Blastocysts scoring ≥3BB on the Gardner grading system (27) were cryopreserved by vitrification using a commercial vitrification kit (Vitrolife, Gothenburg, Sweden). When fresh transfer was performed, embryo transfer took place 3–6 days after oocyte retrieval; supernumerary embryos were vitrified for subsequent use. Cryopreserved embryos were thawed on the morning of transfer using a rapid-thaw protocol (Gothenburg, Sweden).

### 2.5 Embryo transfer and luteal phase support

Cleavage-stage embryos (day 3) or blastocysts (day 5–6) were transferred into the uterine cavity under transabdominal ultrasound guidance, based on individual clinical circumstances. Luteal phase support was initiated on the day of oocyte retrieval for fresh transfer cycles. For patients in whom fresh transfer was deferred, all available embryos were cryopreserved by vitrification with informed consent, and frozen-thawed embryo transfer (FET) was subsequently performed. Endometrial preparation for FET was individualized using either a natural cycle or a hormone replacement protocol. Detailed medication regimens and luteal phase support protocols have been described in previously published studies from our center (28).

### 2.6 Follow-up

Serum hCG was measured 14 days after embryo transfer; a level > 50 IU/L was considered indicative of biochemical pregnancy. Transvaginal ultrasound was performed 28 days post-transfer; clinical pregnancy was defined as the visualization of a gestational sac, whether intrauterine or ectopic. Early pregnancy loss was defined as fetal demise before 12 weeks of gestation, and late pregnancy loss was defined as fetal demise between 12 and 28 completed weeks of gestation.

### 2.7 Outcome measures

#### 2.7.1 Baseline characteristics

Female age, body mass index (BMI), duration and type of infertility, and basal hormonal parameters [FSH, LH, E2, P, prolactin (PRL), testosterone (T)], as well as COS protocol, AFC, and anti-Müllerian hormone (AMH).

#### 2.7.2 Stimulation parameters

Starting Gn dose, total Gn dose, duration of Gn administration, hormonal levels on the day of hCG trigger (E2, LH, P), number of retrieved oocytes, number of metaphase II (MII) oocytes, and MII oocyte rate.

#### 2.7.3 Laboratory parameters

Fertilization method, fertilization rate, number of 2-pronuclei (2PN) zygotes, 2PN rate, cleavage rate, number of available day-3 embryos, number of top-quality embryos, number of blastocysts, and blastocyst formation rate.

#### 2.7.4 Clinical outcomes

Number and type of transferred embryos, endometrial thickness at transfer, cycle type for the first transfer (fresh vs. frozen-thawed), embryo implantation rate, clinical pregnancy rate and live birth rate for fresh cycles, clinical pregnancy rate and live birth rate for FET cycles, ectopic pregnancy rate, miscarriage rate, cumulative pregnancy rate, and cumulative live birth rate.

Key outcome calculations were defined as follows:

1. MII oocyte rate = number of MII oocytes/number of retrieved oocytes × 100%
2. Fertilization rate = number of fertilized oocytes/number of retrieved oocytes × 100%
3. 2PN rate = number of 2PN zygotes/number of MII oocytes × 100%
4. Cleavage rate = total number of cleaved embryos/total number of fertilized oocytes × 100%
5. Embryo implantation rate = number of gestational sacs/total number of transferred embryos × 100%
6. Fresh cycle clinical pregnancy rate = number of clinical pregnancies in fresh cycles/number of fresh transfer cycles × 100%
7. Fresh cycle live birth rate = number of live births in fresh cycles/number of fresh transfer cycles × 100%
8. FET clinical pregnancy rate = number of clinical pregnancies in FET cycles/number of FET cycles × 100%
9. FET live birth rate = number of live births in FET cycles/number of FET cycles × 100%
10. Cumulative pregnancy rate = number of patients achieving first pregnancy (including fresh and FET cycles) within the current retrieval episode/total number of oocyte retrieval cycles × 100%
11. Cumulative live birth rate = number of patients achieving first live birth following a single ovarian stimulation episode/total number of oocyte retrieval cycles × 100%

### 2.8 Follow-up and CLBR ascertainment

The follow-up duration for the primary outcome (CLBR) was determined by cycle completion rather than a fixed temporal cutoff. Specifically, follow-up continued until a patient had transferred all available embryos (including both fresh and frozen-thawed embryos) generated from the single oocyte retrieval cycle, or until the first live birth was achieved, whichever occurred first. The analysis was conducted on an intention-to-treat (ITT) basis, including all patients who underwent oocyte retrieval in the denominator. Patients who had remaining cryopreserved embryos but voluntarily discontinued subsequent embryo transfers for non-medical reasons were classified as not achieving a live birth; all such patients completed a formal embryo disposal process with written informed consent in accordance with institutional ethical regulations.

### 2.9 OMA measurement

OMA diameter was determined by TVUS performed by experienced sonographers (1–2 per examination); MRI was used for confirmation in cases of complex or multilocular cysts. The measurement time point was standardized as the most recent ultrasound within 1–3 months prior to COS initiation. For patients with bilateral OMA, the maximum diameter of the larger cyst was used for group classification, and laterality (unilateral vs. bilateral) was recorded as a separate covariate. Sonographers were not blinded to treatment allocation, as all measurements were obtained as part of routine clinical care.

### 2.10 Statistical analysis

All statistical analyses were performed using Python 3.12 (Python Software Foundation, Wilmington, DE, USA). Missing data were minimal across all key variables. In the comparison between Groups B and C (n = 278), AMH values were missing for 10 patients (3.6%) and AFC values were missing for 3 patients (1.1%), resulting in 266 patients (95.7%) with complete covariate data available for propensity score analysis. Missing AMH data occurred primarily in patients treated during 2016-2017, when AMH testing had not yet been routinely incorporated into the pre-IVF evaluation at our center. Missing AFC data resulted from incomplete documentation in ultrasound reports. These data were considered to be missing at random (MAR), as the missingness was attributable to temporal changes in clinical protocols rather than patient characteristics or outcomes. Given the low proportion of missing data (<5% for all variables), complete case analysis was used for all multivariable analyses, which is generally considered appropriate when the missing data proportion is minimal and the missingness mechanism is unrelated to outcomes (9, 29). A detailed summary of missing data by variable and study group is provided in **Supplementary Table 11**.

Continuous variables conforming to a normal distribution are expressed as mean ± standard deviation (x̄ ± s) and were compared between groups using the independent-samples t-test. Non-normally distributed continuous variables are presented as median (25^th^-75th percentile) [M (Q1, Q3)] and were compared using the Mann-Whitney U test or Kruskal-Wallis test as appropriate. Categorical variables are expressed as frequencies and proportions (%), with between-group comparisons performed using the chi-square (χ²) test; Fisher’s exact test was applied when the expected cell frequency was less than 1. Multiple linear regression analysis was employed to investigate the independent effects of OMA size and surgical status on the number of retrieved oocytes after adjusting for potential confounders. Multivariable logistic regression analysis was performed to identify factors independently associated with cumulative live birth rate after adjusting for confounders. A two-tailed P-value < 0.05 was considered statistically significant. The primary outcome was cumulative live birth rate, which was the sole endpoint for confirmatory hypothesis testing. All secondary outcomes-including number of retrieved oocytes, embryological parameters, and per-transfer pregnancy rates-were considered exploratory and are reported descriptively to characterize the biological cascade through which surgical intervention may affect IVF efficiency. No adjustment for multiple comparisons was applied to secondary outcome analyses, as these were not intended to test independent hypotheses but rather to elucidate the mechanistic pathway from surgical ovarian damage to clinical outcomes. Accordingly, secondary outcome results should be interpreted with appropriate caution and regarded as hypothesis-generating.

To address potential confounding in the comparison between non-surgical (Group B) and surgical (Group C) OMA ≥4 cm cohorts, propensity score matching (PSM) was performed. Propensity scores were estimated using logistic regression with the following covariates: age, BMI, AMH, AFC, duration of infertility, type of infertility (primary vs. secondary), and OMA laterality (unilateral vs. bilateral). One-to-one nearest-neighbor matching without replacement was performed using a caliper width of 0.2 standard deviations of the logit of the propensity score. Covariate balance was assessed using standardized mean differences (SMD), with SMD <0.1 indicating adequate balance. As a sensitivity analysis, inverse probability of treatment weighting (IPTW) with stabilized, truncated weights was also performed to confirm the robustness of PSM findings.

As this was a retrospective cohort study utilizing existing clinical data, no *a priori* sample size calculation was performed. Instead, all eligible patients meeting the inclusion and exclusion criteria during the study period were enrolled. *Post-hoc* power analysis was conducted to evaluate the statistical power of key comparisons given the observed sample sizes and effect sizes. Power calculations were performed using the two-proportion z-test with α = 0.05.

Exploratory subgroup analyses were performed for the comparison between Groups B and C, stratified by age (<35 vs. ≥35 years), AMH level (normal ≥1.1 vs. low <1.1 ng/mL), embryo type (cleavage-stage vs. blastocyst), and OMA laterality (unilateral vs. bilateral). Interaction terms were included in logistic regression models to test for effect modification; a P-value for interaction <0.05 was considered indicative of significant heterogeneity. Due to the exploratory nature and multiple comparisons, these subgroup results should be interpreted with caution.

As an additional sensitivity analysis, bilateral OMA status was added as a covariate to all multivariable regression models to assess whether OMA laterality confounded the primary findings. A change in the effect estimate of >10% was considered indicative of meaningful confounding.

The marked imbalance in group sizes between the non-surgical (Group B, n=47) and surgical (Group C, n=231) OMA ≥4 cm cohorts reflects prevailing clinical practice at our institution, where laparoscopic cystectomy has been the standard recommendation for OMA ≥4 cm in accordance with national guidelines. This real-world distribution, while limiting statistical power for some comparisons, provides a pragmatic assessment of outcomes under current care patterns and underscores the clinical relevance of the research question—namely, whether the guideline-recommended surgical approach yields superior reproductive outcomes. Detailed results for all secondary outcomes, subgroup analyses, sensitivity analyses, and missing data summaries are provided in the Supplementary Tables (**Supplementary Tables 1**–**S12**) and Supplementary Figures (**Supplementary Figure 1**).

This study was designed within a superiority testing framework to evaluate whether prior cystectomy is associated with improved CLBR compared with direct IVF. It was not designed or powered as a non-inferiority or equivalence trial; accordingly, failure to reject the null hypothesis of no difference should not be interpreted as evidence that the two management strategies produce equivalent reproductive outcomes.

### 2.11 Ethical approval

This study was approved by the Reproductive Medicine Ethics Committee of Henan Provincial People’s Hospital (Approval Number: SYSZ-LL-2019012410) and was conducted in accordance with the Declaration of Helsinki for Medical Research involving Human Participants. Due to the retrospective nature of the study and the use of de-identified clinical data, the requirement for individual written informed consent was waived by the ethics committee. The study was reported in accordance with the Strengthening the Reporting of Observational Studies in Epidemiology (STROBE) guidelines for cohort studies (30).

## 3 Results

### 3.1 Baseline characteristics of the study population

A total of 394 infertile patients with OMA undergoing their first IVF cycle were included and stratified into three groups: Group A (non-surgical OMA <4 cm, n=116), Group B (non-surgical OMA ≥4 cm, n=47), and Group C (surgical OMA ≥4 cm, n=231). Patient selection and group allocation are illustrated in **Figure 1**.

The three groups were generally well-matched with respect to age, BMI, duration and type of infertility, and most basal hormonal parameters (**Table 1**). Notable between-group differences were observed in the following: primary infertility was more prevalent in Group B than Group A (65.96% vs. 43.10%, P = 0.008); basal testosterone was higher in Group B than Group A (median 0.28 vs. 0.19 ng/mL, P = 0.042); basal LH was higher in Group B than Group C (median 5.54 vs. 4.66 IU/L, P = 0.005); and serum AMH was significantly lower in Group C than in both Group B (median 1.98 vs. 3.16 ng/mL, P = 0.001) and Group A (median 1.98 vs. 2.87 ng/mL, P<0.001), indicating that patients who had undergone prior cystectomy had substantially reduced ovarian reserve compared with non-surgically managed patients. Serum AMH was comparable between Groups A and B (2.87 vs. 3.16 ng/mL, P = 0.425).

**Table 1:** Baseline characteristics of infertile patients with ovarian endometrioma stratified by management strategy and cyst size.

Variable	Group A (n=116) non-surgical OMA <4 cm	Group B (n=47) non-surgical OMA ≥4 cm	Group C (n=231) surgical OMA ≥4 cm	P value (A vs B)	P value (B vs C)
Age (years)	32.00 (29.00, 33.25)	31.00 (29.00, 33.00)	31.00 (29.00, 33.50)	0.600	0.912
BMI (kg/m²)	21.03 (19.45, 22.88)	21.26 (20.00, 22.95)	21.60 (19.98, 23.97)	0.530	0.352
Duration of infertility (years)	3.00 (2.00, 5.00)	2.00 (1.50, 4.00)	3.00 (2.00, 5.00)	0.100	0.362
Type of infertility				0.008*	0.122
Primary infertility	43.10% (50/116)	65.96% (31/47)	53.68% (124/231)		
Secondary infertility	56.90% (66/116)	34.04% (16/47)	46.32% (107/231)		
Basal FSH (IU/L)	6.59 (5.64, 8.07)	6.63 (6.01, 7.33)	6.96 (5.87, 8.51)	0.823	0.230
Basal LH (IU/L)	5.18 (3.83, 6.60)	5.54 (4.81, 6.69)	4.66 (3.52, 6.04)	0.171	0.005*
Basal E2 (pg/mL)	43.02 (33.47, 53.06)	39.97 (32.11, 52.23)	44.79 (33.77, 59.55)	0.516	0.134
Basal P (ng/mL)	0.36 (0.19, 0.53)	0.33 (0.23, 0.46)	0.295 (0.190, 0.515)	0.950	0.502
Basal PRL (ng/mL)	15.70 (12.23, 20.24)	17.41 (13.74, 22.89)	15.50 (11.61, 20.10)	0.135	0.070
Basal T (ng/mL)	0.19 (0.15, 0.29)	0.28 (0.17, 0.35)	0.24 (0.14, 0.32)	0.042*	0.189
AMH (ng/mL)	2.87 (1.68, 4.25)	3.16 (1.97, 4.61)	1.98 (1.17, 3.34)	0.425	0.001*
Basal AFC (count)	9.00 (6.00, 13.00)	8.00 (7.00, 11.00)	8.00 (5.00, 11.00)	0.379	0.570
COS protocol				0.152†	0.086†
Ultra-long protocol	0.86% (1/116)	6.38% (3/47)	1.30% (3/231)		
Long protocol	86.21% (101/116)	78.72% (37/47)	73.16% (169/231)		
Antagonist protocol	8.62% (10/116)	12.77% (6/47)	18.61% (43/231)		
Non-downregulation protocol	4.31% (5/116)	2.13% (1/47)	6.93% (16/231)		

Data are presented as median (interquartile range) for continuous variables and n (%) for categorical variables. Statistical comparisons were performed using the Mann-Whitney U test for continuous variables and the chi-square test or Fisher’s exact test for categorical variables, as appropriate. Group A: non-surgical management of OMA <4 cm; Group B: non-surgical management of OMA ≥4 cm; Group C: laparoscopic cystectomy for OMA ≥4 cm performed prior to IVF. *P < 0.05; ^†^Fisher’s exact test. Bold values indicate statistically significant results (P < 0.05).

AFC, antral follicle count; AMH, anti-Müllerian hormone; BMI, body mass index; COS, controlled ovarian stimulation; E2, estradiol; FSH, follicle-stimulating hormone; IVF, *in vitro* fertilization; LH, luteinizing hormone; OMA, ovarian endometrioma; P, progesterone; PRL, prolactin; T, testosterone.

### 3.2 Impact of OMA size on IVF outcomes in non-surgically managed patients (Group A vs. Group B)

In patients with OMA managed non-surgically, cyst size (<4 cm vs. ≥4 cm) had no significant impact on IVF outcomes. COS parameters, including starting Gn dose, total Gn consumption, stimulation duration, trigger-day hormones, and number of retrieved oocytes, were comparable between Groups A and B (**Supplementary Table 1**). Embryological outcomes—including fertilization rate, 2PN count, cleavage rate, number of available Day-3 embryos, top-quality embryos, and blastocyst formation rate—were also similar between groups, with the exception of a marginally higher 2PN rate in Group B (77.98% vs. 72.04%, P = 0.020), which likely reflects the higher MII oocyte maturation rate observed in that group rather than any qualitative superiority (**Supplementary Table 1**).

**Figure 2:**
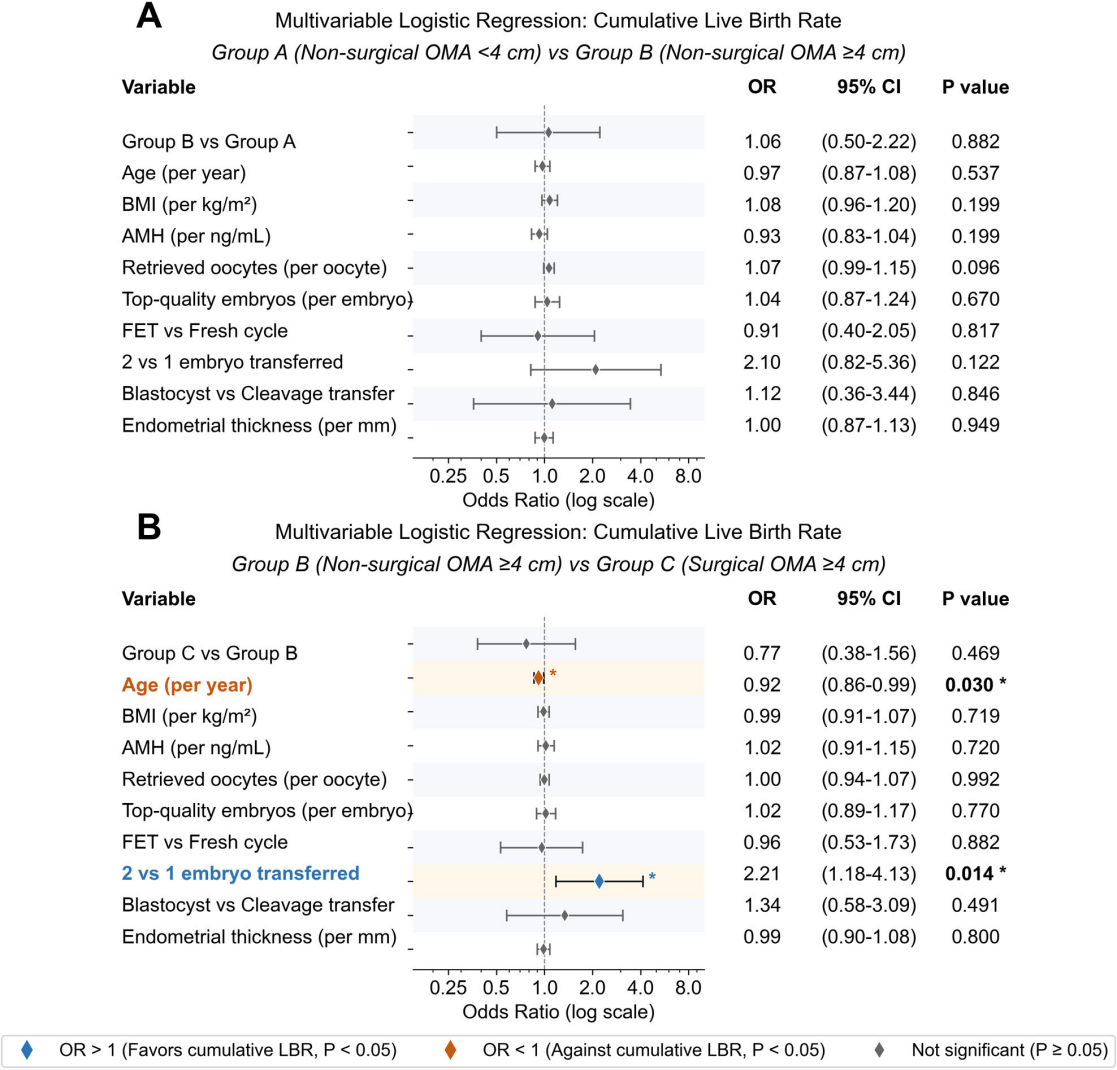
Multivariable logistic regression analysis of independent predictors of cumulative live birth rate. **(A)** Group A (non-surgical OMA <4 cm, n = 116) versus Group B (non-surgical OMA ≥4 cm, n = 47) (**Supplementary Table 8**). Diamonds represent point estimates of the adjusted odds ratio (OR); horizontal lines represent 95% confidence intervals (CI). No variable reached statistical significance in this model. OMA size (Group B vs. Group A: OR = 1.06, 95% CI: 0.50–2.22, P = 0.882) was not an independent predictor of cumulative live birth rate after adjustment for confounders. The widest confidence interval was observed for the transfer of two versus one embryo (OR = 2.10, 95% CI: 0.82–5.36, P = 0.122), reflecting the limited statistical power of this comparison given the small sample size of Group B All variables were adjusted for simultaneously in the model. **(B)** Group B (non-surgical OMA ≥4 cm, n = 47) versus Group C (surgical OMA ≥4 cm, n = 231) (**Table 4**). Two variables reached statistical significance: advancing age was independently associated with a reduced probability of cumulative live birth (OR = 0.92 per year, 95% CI: 0.86–0.99, P = 0.030), and the transfer of two embryos versus one was independently associated with an increased probability of cumulative live birth (OR = 2.21, 95% CI: 1.18–4.13, P = 0.014). Surgical status (Group C vs. Group B) was not an independent predictor of cumulative live birth rate (OR = 0.77, 95% CI: 0.38–1.56, P = 0.469). Significant rows are highlighted in light yellow. *P < 0.05. In the legend, a blue diamond indicates OR > 1 (favors cumulative LBR, P < 0.05), an orange diamond indicates OR < 1 (against cumulative LBR, P < 0.05), and a gray diamond indicates not significant (P ≥0.05). OR, odds ratio; CI, confidence interval; LBR, live birth rate; FET, frozen-thawed embryo transfer; OMA, ovarian endometrioma; AMH, anti-Müllerian hormone; BMI, body mass index.

No statistically significant differences in clinical pregnancy outcomes were observed across measured endpoints (**Supplementary Table 2**). Specifically, cumulative live birth rate was 58.62% (68/116) in Group A and 59.57% (28/47) in Group B (P = 0.911). Multiple linear regression confirmed that OMA size was not independently associated with retrieved oocyte number (β=0.29, P = 0.954; **Supplementary Table 3** Univariable logistic regression showed that among all variables examined, only the number of retrieved oocytes reached statistical significance (OR = 1.06, 95% CI: 1.01–1.12, P = 0.043), while OMA size was not a significant predictor (OR = 1.04, 95% CI: 0.52–2.07, P = 0.911; **Supplementary Table 5**). Multivariable logistic regression further confirmed that OMA size was not an independent predictor of cumulative live birth rate after adjustment for confounders (Group B vs. Group A: OR = 1.06, 95% CI: 0.50–2.22, P = 0.882; **Supplementary Table 8**; **Figure 2A**). However, the wide confidence interval (OR: 0.50–2.22) and limited sample size preclude definitive conclusions regarding equivalence between the two groups.

#### 3.2.1 Statistical power considerations

With the current sample sizes (n=116 vs. n=47) and the observed CLBR of 58.62% versus 59.57% (Δ=0.95 percentage points), the 95% confidence interval for the absolute difference ranged from −15.7% to +17.6%, indicating that this comparison cannot exclude clinically meaningful differences in either direction. The minimum detectable difference with 80% power at α=0.05 was 23.8 percentage points, confirming that this comparison was substantially underpowered. These results should therefore be regarded as indicating no evidence of a large effect of OMA size on CLBR rather than evidence of equivalence. These findings should therefore be interpreted as absence of evidence for a large effect of OMA size rather than definitive evidence of equivalence. Impact of Prior Cystectomy on Ovarian Reserve and IVF Efficiency (Group B vs. Group C).

### 3.3 Controlled ovarian stimulation parameters and embryological outcomes

**Figure 3:**
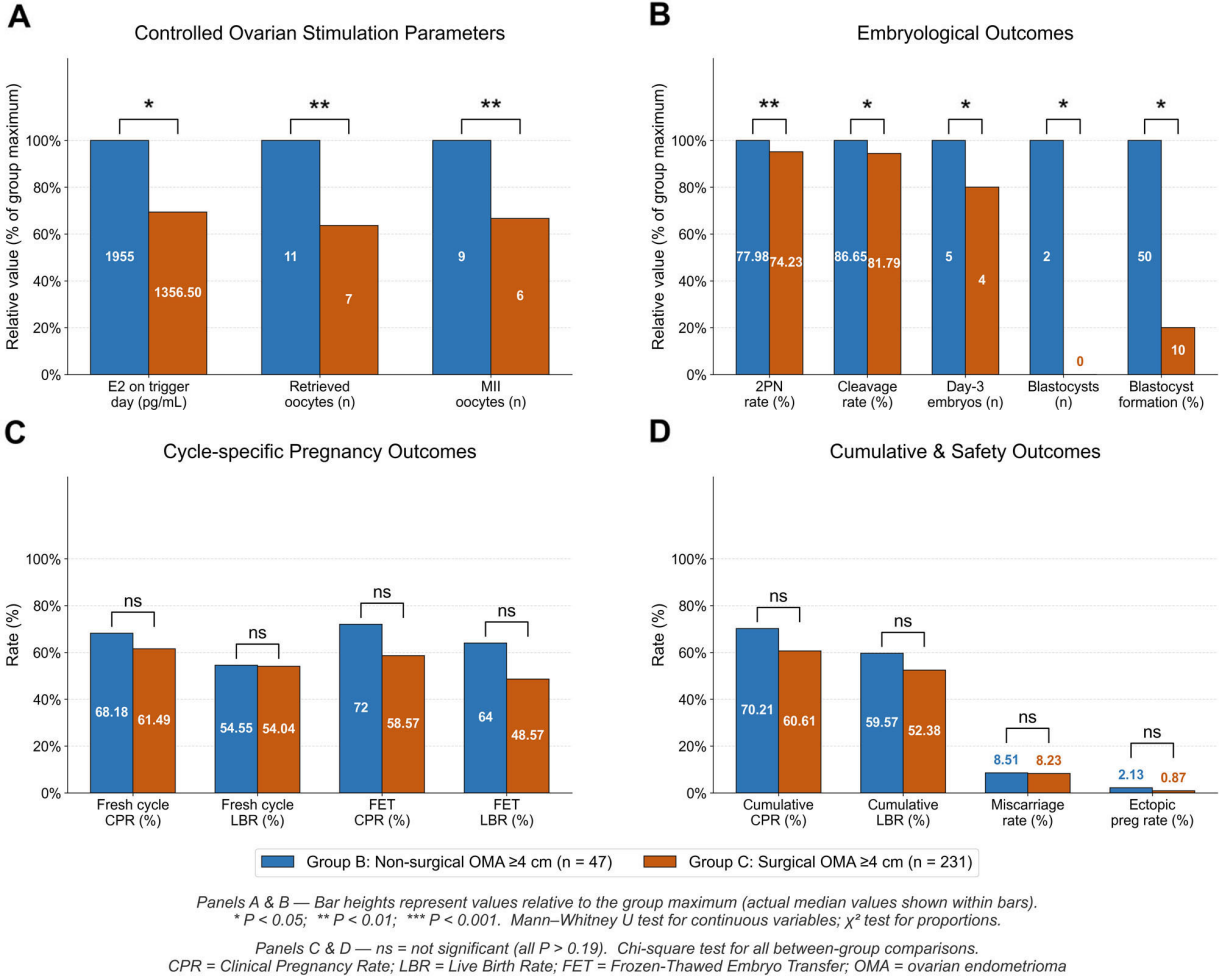
Comparison of IVF outcomes between non-surgical management (Group B, n = 47) and prior laparoscopic cystectomy (Group C, n = 231) in patients with ovarian endometrioma ≥4 cm. **(A)** Controlled ovarian stimulation parameters: serum estradiol on trigger day, number of retrieved oocytes, and number of MII oocytes. **(B)** Embryological outcomes: 2PN rate, cleavage rate, available Day-3 embryo count, blastocyst count, and blastocyst formation rate. In Panels **(A, B)**, bar heights represent values relative to the group maximum for each variable; actual median values are shown within bars. Statistical comparisons were performed using the Mann–Whitney U test for continuous variables and the chi-square test for proportions. *P < 0.05; **P < 0.01. **(C)** Cycle-specific pregnancy outcomes: clinical pregnancy rate and live birth rate for fresh and frozen-thawed embryo transfer cycles. **(D)** Cumulative and safety outcomes: cumulative pregnancy rate, cumulative live birth rate, miscarriage rate, and ectopic pregnancy rate. In Panels **(C, D)**, bar heights represent actual percentages; all between-group comparisons were non-significant (all P > 0.19; chi-square test). ns, not significant. 2PN, two pronuclei; CPR, clinical pregnancy rate; E2, estradiol; FET, frozen-thawed embryo transfer; LBR, live birth rate; MII, metaphase II; OMA, ovarian endometrioma.

Among the 278 patients with OMA ≥4 cm, those who had undergone prior laparoscopic cystectomy (Group C, n=231) demonstrated significantly inferior ovarian response compared with non-surgically managed patients (Group B, n=47). Serum E2 on the day of trigger was significantly lower in Group C (median 1,356.50 vs. 1,955.00 pg/mL, P = 0.035). More notably, the total number of retrieved oocytes was substantially reduced in Group C (median 7.00 vs. 11.00, P = 0.001), as was the number of MII oocytes (median 6.00 vs. 9.00, P = 0.002; **Table 2**; **Figure 3A**).

**Table 2:** Controlled ovarian stimulation parameters and embryological outcomes in patients with ovarian endometrioma ≥4 cm: non-surgical management (Group B) versus prior laparoscopic cystectomy (Group C).

Variable	Group B (n=47) non-surgical OMA ≥4 cm	Group C (n=231) surgical OMA ≥4 cm	Z/χ² value	P value
Controlled ovarian stimulation parameters				
Starting Gn dose (IU)	162.50 (125.00, 225.00)	200.00 (150.00, 225.00)	−0.981	0.332
Total Gn dose (IU)	2325.00 (1800.00, 2775.00)	2250.00 (1800.00, 2962.50)	0.025	0.992
Duration of Gn stimulation (days)	11.00 (10.00, 12.00)	10.00 (8.00, 12.00)	1.606	0.122
E2 on trigger day (pg/mL)	1955.00 (1057.75, 3000.00)	1356.50 (845.35, 2040.25)	−2.214	0.035*
LH on trigger day (IU/L)	1.12 (0.68, 2.07)	1.56 (0.65, 2.97)	−1.552	0.122
P on trigger day (ng/mL)	0.69 (0.41, 1.03)	0.61 (0.39, 0.91)	−0.535	0.603
Retrieved oocytes (count)	11.00 (6.00, 15.00)	7.00 (5.00, 11.00)	3.245	0.001*
MII oocytes (count)	9.00 (6.00, 12.50)	6.00 (4.00, 10.00)	3.126	0.002*
MII oocyte rate (%)	87.43% (445/509)	86.30% (1587/1839)	0.444	0.505
Embryological outcomes				
Fertilization method				0.763
Conventional IVF	87.23% (41/47)	90.04% (208/231)		
ICSI	12.77% (6/47)	9.96% (23/231)		
Fertilization rate (%)	80.94% (412/509)	79.45% (1461/1839)	0.552	0.457
2PN count (per patient)	6.00 (3.50, 10.00)	4.00 (2.00, 7.00)	2.612	0.113
2PN rate (%)	77.98% (347/445)	74.23% (1178/1587)	3.292	0.001*
Cleavage rate (%)	86.65% (357/412)	81.79% (1195/1461)	5.339	0.021*
Available Day-3 embryos (count)	5.00 (3.00, 8.00)	4.00 (2.00, 6.00)	−2.743	0.012*
Top-quality embryos (count)	2.00 (0.00, 3.50)	1.00 (0.00, 3.00)	1.173	0.242
Blastocyst count (per patient)	2.00 (0.00, 4.00)	0.00 (0.00, 3.00)	2.563	0.015*
Blastocyst formation rate (%)	50.00 (0.00, 69.05)	10.00 (0.00, 62.26)	2.153	0.035*

Data are presented as median (interquartile range) for continuous variables and % (n/N) for rates. Statistical comparisons were performed using the Mann-Whitney U test for continuous variables and the chi-square test for categorical variables. Group B: non-surgical management of OMA ≥4 cm; Group C: laparoscopic cystectomy for OMA ≥4 cm performed prior to IVF. *P < 0.05. Bold values indicate statistically significant results (P < 0.05).

2PN, two pronuclei; E2, estradiol; Gn, gonadotropin; ICSI, intracytoplasmic sperm injection; IVF, *in vitro* fertilization; LH, luteinizing hormone; MII, metaphase II; OMA, ovarian endometrioma; P, progesterone.

Multiple linear regression analysis confirmed that prior cystectomy was independently associated with a reduction in oocyte yield after adjustment for age, BMI, AMH, AFC, basal FSH, Gn parameters, and COS protocol (β=−1.92, 95% CI: −3.48 to −0.39, P = 0.015), indicating that surgery was associated with retrieval of approximately 2 fewer oocytes per cycle on average, independent of baseline ovarian reserve parameters (**Supplementary Table 4**).

Consistent with the reduced oocyte yield, Group C demonstrated significantly inferior embryological outcomes across multiple parameters: lower 2PN rate (74.23% vs. 77.98%, P = 0.001), lower cleavage rate (81.79% vs. 86.65%, P = 0.021), fewer available Day-3 embryos (median 4.00 vs. 5.00, P = 0.012), fewer blastocysts (median 0.00 vs. 2.00, P = 0.015), and lower blastocyst formation rate (10.00% vs. 50.00%, P = 0.035; **Table 2**; **Figure 3B**). These results demonstrate that prior cystectomy not only reduces the number of retrieved oocytes but also compromises downstream embryological efficiency.

### 3.4 Impact of prior cystectomy on cumulative live birth rate (Group B vs. Group C)

#### 3.4.1 Clinical pregnancy outcomes

Despite the marked differences in ovarian response and embryological parameters, no statistically significant differences in clinical pregnancy outcomes were identified between Groups B and C (**Table 3**; **Figures 3C, D**). No significant differences were identified in clinical pregnancy rate in fresh cycles (68.18% vs. 61.49%, P = 0.543), live birth rate in fresh cycles (54.55% vs. 54.04%, P = 0.947), clinical pregnancy rate in FET cycles (72.00% vs. 58.57%, P = 0.245), live birth rate in FET cycles (64.00% vs. 48.57%, P = 0.197), cumulative pregnancy rate (70.21% vs. 60.61%, P = 0.220), or cumulative live birth rate (59.57% vs. 52.38%, P = 0.370). Two parameters differed significantly: the type of first transfer cycle (fresh vs. FET, P = 0.003) and the type of transferred embryos (cleavage-stage vs. blastocyst, P = 0.023), both reflecting the lower number of available blastocysts in Group C.

**Table 3:** Clinical pregnancy outcomes: Group B vs Group C.

Variable	Group B (n=47)	Group C (n=231)	Z/x² value	P value
Endometrial thickness at transfer (mm)	10.00 (9.00, 12.00)	11.00 (9.00, 13.00)	-0.887	0.38
First transfer cycle type (%)			9.101	0.003*
Fresh cycle	46.81% (22/47)	69.70% (161/231)		
Frozen-thawed cycle	53.19% (25/47)	30.30% (70/231)		
Number of transferred embryos (%)			0.312	0.580
1 embryo	46.81% (22/47)	42.42% (98/231)		
2 embryos	53.19% (25/47)	57.58% (133/231)		
Type of transferred embryos (%)			5.188	0.023*
Cleavage-stage	65.96% (31/47)	80.95% (187/231)		
Blastocyst	34.04% (16/47)	19.05% (44/231)		
Embryo implantation rate (%)	54.17% (39/72)	48.08% (175/364)	0.896	0.354
Fresh cycle clinical pregnancy rate (%)	68.18% (15/22)	61.49% (99/161)	0.373	0.543
Fresh cycle live birth rate (%)	54.55% (12/22)	54.04% (87/161)	0.002	0.947
FET clinical pregnancy rate (%)	72.00% (18/25)	58.57% (41/70)	1.414	0.245
FET live birth rate (%)	64.00% (16/25)	48.57% (34/70)	1.766	0.197
Ectopic pregnancy rate (%)	2.13% (1/47)	0.87% (2/231)	0.583	0.445
Miscarriage rate (%)	8.51% (4/47)	8.23% (19/231)	0.004	0.956
Cumulative pregnancy rate (%)	70.21% (33/47)	60.61% (140/231)	1.538	0.220
Cumulative live birth rate (%)	59.57% (28/47)	52.38% (121/231)	0.810	0.370

Data are presented as n (%). Clinical pregnancy was defined as the presence of an intrauterine gestational sac with fetal cardiac activity on transvaginal ultrasound at 6–7 weeks of gestation. Early miscarriage was defined as pregnancy loss before 12 weeks of gestation. Cumulative outcomes include all fresh and frozen embryo transfer cycles until first live birth or exhaustion of all available embryos within the study period. *P < 0.05. Bold values indicate statistically significant results (P < 0.05).

CI, confidence interval; FET, frozen embryo transfer; IQR, interquartile range.

#### 3.4.2 Regression analysis

Univariable logistic regression identified age (OR = 0.93, P = 0.031), starting Gn dose (OR = 0.99, P = 0.005), and number of transferred embryos (2 vs. 1: OR = 1.74, P = 0.024) as significant predictors of cumulative live birth rate. Surgical status was not a significant univariable predictor (OR = 0.75, P = 0.368; **Supplementary Table 6**).

Multivariable logistic regression confirmed that surgical status was not an independent predictor of cumulative live birth rate after adjustment for confounders (Group C vs. Group B: OR = 0.77, 95% CI: 0.38–1.56, P = 0.469; **Table 4**; **Figure 2B**).

**Table 4:** Multivariable logistic regression: independent predictors of cumulative live birth rate (Group B vs Group C).

Variable	β	OR (95% CI)	P value
Group (reference: Group B)			
Group C	-0.26	0.77 (0.38-1.56)	0.469
Age	-0.08	0.92 (0.86-0.99)	0.030*
BMI	-0.02	0.99 (0.91-1.07)	0.719
AMH	0.02	1.02 (0.91-1.15)	0.720
Retrieved oocytes	0.00	1.00 (0.94-1.07)	0.992
Top-quality embryos	0.02	1.02 (0.89-1.17)	0.770
First transfer cycle type (reference: fresh cycle)			
Frozen-thawed cycle	-0.04	0.96 (0.53-1.73)	0.882
Number of transferred embryos (reference: 1)			
2 embryos	0.79	2.21 (1.18-4.13)	0.014*
Type of transferred embryos (reference: cleavage-stage)			
Blastocyst	0.29	1.34 (0.58-3.09)	0.491
Endometrial thickness at transfer	-0.01	0.99 (0.90-1.08)	0.800

Multivariable logistic regression analysis with cumulative live birth as the dependent variable. The model included variables with P < 0.10 in univariable analysis and clinically relevant confounders (age, BMI, AMH). Group comparison uses Group B (non-surgical) as the reference category. The Hosmer-Lemeshow test was used to assess goodness-of-fit. The area under the receiver operating characteristic curve (AUC-ROC) is reported to assess discriminative ability.

AFC, antral follicle count; AMH, anti-Müllerian hormone; AUC, area under the curve; BMI, body mass index; CI, confidence interval; OMA, ovarian endometrioma; OR, odds ratio; ROC, receiver operating characteristic. *P < 0.05. Bold values indicate statistically significant results (P < 0.05).

#### 3.4.3 Propensity score matching analysis

To further address potential selection bias, propensity score matching was performed. Of the 278 patients with OMA ≥4 cm (Group B, n=47; Group C, n=231), 12 were excluded from the PSM analysis due to missing covariate data (AMH: n=10; AFC: n=2), yielding 266 patients (Group B, n=45; Group C, n=221) with complete data for propensity score estimation. Of 266 patients with complete covariate data, 41 matched pairs were successfully identified. After matching, cumulative live birth rates remained comparable between Groups B and C (60.98% vs. 51.22%; OR = 0.67, 95% CI: 0.28–1.61, P = 0.374; **Supplementary Table 7**; **Table 5**). IPTW analysis yielded consistent results (OR = 0.77, 95% CI: 0.40–1.50, P = 0.448). Retrieved oocyte counts remained significantly lower in Group C after both PSM (median 7 vs. 10, β=−4.02, P = 0.001) and IPTW adjustment (β=−2.72, P = 0.002).

**Table 5:** Propensity score matching analysis: comparison of outcomes between Group B and Group C.

Analysis	Outcome	Group B	Group C	Effect estimate	95% CI	P
Before PSM	Cumulative live birth rate	27/45 (60.00%)	118/221 (53.39%)	OR = 0.76	0.40–1.47	0.418
After PSM (1:1)	Cumulative live birth rate	25/41 (60.98%)	21/41 (51.22%)	OR = 0.67	0.28–1.61	0.374
IPTW	Cumulative live birth rate	59.96% (weighted)	53.67% (weighted)	OR = 0.77	0.40–1.50	0.448
Before PSM	Retrieved oocytes	10.0 (6.0, 15.0)	7.0 (5.0, 11.0)	β = −2.58	−4.25 to −0.91	0.004*
After PSM (1:1)	Retrieved oocytes	10.0 (6.0, 15.0)	7.0 (3.0, 9.0)	β = −4.02	−6.39 to −1.66	0.001**
IPTW	Retrieved oocytes	Weighted mean	Weighted mean	β = −2.72	−4.41 to −1.02	0.002**

Outcomes were compared between the non-surgical (Group B) and surgical (Group C) management groups using three analytical approaches: (1) unadjusted analysis before PSM, (2) matched-pair analysis after 1:1 PSM, and (3) IPTW-weighted analysis. For cumulative live birth rate (binary outcome), logistic regression was used to estimate odds ratios (OR), with Group B (non-surgical) as the reference category; therefore, OR <1 indicates higher odds of live birth in Group C (surgical). For retrieved oocyte number (continuous outcome), linear regression was used to estimate the mean difference (β coefficient), with Group B as the reference; therefore, β <0 indicates fewer oocytes retrieved in Group C. Retrieved oocytes are presented as median (Q1, Q3). Statistical significance: *P < 0.05; **P < 0.01. The sample sizes in this table (n=45/221 before PSM) differ from those in **Tables 1**–**3** (n=47/231) because 12 patients with missing AMH or AFC values were excluded from the propensity score analysis, which required complete covariate data. Bold values indicate statistically significant results (P < 0.05).

The wide confidence intervals for all analytical approaches (multivariable regression: 0.38–1.56; PSM: 0.28–1.61; IPTW: 0.40–1.50) reflect the limited precision of these estimates and are compatible with both clinical equivalence and a clinically meaningful detriment of cystectomy on CLBR.

#### 3.4.4 Subgroup analyses

Exploratory subgroup analyses assessed whether the effect of surgical status on cumulative live birth rate varied across clinically relevant subgroups (**Supplementary Table 9**; **Supplementary Figure 1**). No significant associations were observed in patients aged <35 years (OR = 0.73, P = 0.370), those with normal AMH (OR = 0.76), or in unilateral (OR = 0.75) and bilateral OMA subgroups (OR = 0.72). No significant interaction effects were detected (all P-interaction >0.05).

#### 3.4.5 Sensitivity analysis

Bilateral OMA status was added as an additional covariate to assess potential confounding (**Supplementary Table 10**). The effect estimate for cumulative live birth rate remained virtually unchanged (original OR = 0.77; adjusted OR = 0.77; change=+0.3%). Bilateral OMA status itself was not significantly associated with cumulative live birth rate (P>0.2).

E-value analysis was performed to quantify the robustness of the primary findings to potential unmeasured confounding (**Supplementary Table 12**). For the comparison of CLBR between Groups B and C, the E-value for the point estimate (OR = 0.77) was 1.92, indicating that an unmeasured confounder would need to be associated with both surgical management and live birth by a risk ratio of at least 1.92-fold each, above and beyond the measured covariates, to fully explain away the observed association. The E-value for the confidence interval limit closest to the null was 1.0, reflecting that the 95% CI already includes the null and that even minimal unmeasured confounding could shift the estimate to unity.

### 3.5 Independent predictors of cumulative live birth rate

Across both comparisons, patient age and number of transferred embryos emerged as the primary independent predictors of cumulative live birth rate (**Table 4**). In the OMA ≥4 cm cohort (Groups B and C), advancing age was independently associated with reduced probability of live birth (OR = 0.92 per year, 95% CI: 0.86–0.99, P = 0.030), while transfer of two embryos compared with one significantly increased the probability of live birth (OR = 2.21, 95% CI: 1.18–4.13, P = 0.014). Neither surgical status (OR = 0.77, P = 0.469) nor OMA size in the non-surgical cohort (OR = 1.06, P = 0.882) independently predicted cumulative live birth rate after multivariable adjustment.

## 4 Discussion

### 4.1 Principal findings

The present retrospective cohort study of 394 infertile patients with OMA yielded three principal findings with direct clinical relevance. First, in non-surgically managed patients, OMA size (<4 cm vs. ≥4 cm) did not independently predict oocyte yield, embryological outcomes, or cumulative live birth rate, challenging the assumption that larger cyst size per se constitutes an indication for pre-IVF surgical intervention. Second, among patients with OMA ≥4 cm, prior laparoscopic cystectomy was associated with significantly reduced ovarian reserve (AMH 1.98 vs. 3.16 ng/mL), fewer retrieved oocytes (median 7 vs. 11), and impaired embryological outcomes—yet cumulative live birth rates did not differ significantly between surgical and non-surgical management (52.4% vs. 59.6%, P = 0.370), although the confidence intervals were wide. Third, patient age and number of transferred embryos—rather than OMA size or surgical status—emerged as the only independent predictors of cumulative live birth rate. These findings support a paradigm shift toward individualized, fertility-preserving management strategies for infertile patients with OMA.

### 4.2 Impact of prior cystectomy on cumulative live birth rate

The central finding of this study is that no statistically significant improvement in cumulative live birth rate was detected following laparoscopic cystectomy prior to IVF in patients with OMA ≥4 cm. Despite significantly fewer retrieved oocytes, fewer embryos, and lower blastocyst formation rates in the surgical group, the difference in cumulative live birth rate between surgical and non-surgical management did not reach statistical significance (52.4% vs. 59.6%; adjusted OR = 0.77, 95% CI: 0.38–1.56, P = 0.469). Propensity score matching and IPTW analyses confirmed the robustness of this finding.

This apparent paradox—whereby cystectomy impairs the quantitative efficiency of IVF without reducing the cumulative probability of live birth—warrants mechanistic consideration. Several complementary explanations may account for this dissociation between surrogate and definitive endpoints. First, in the era of universal vitrification and elective frozen-thawed embryo transfer (FET), the cumulative utilization of all embryos generated from a single stimulation cycle serves as a buffer against reduced per-cycle oocyte yield (31, 32). Even when fewer oocytes are retrieved, patients retain the opportunity to transfer cryopreserved embryos in subsequent FET cycles, thereby progressively accumulating their probability of live birth over time. This “cumulative FET effect” may substantially attenuate the impact of reduced oocyte numbers on the ultimate reproductive outcome, particularly when embryo quality is preserved. Second, cystectomy may disproportionately affect oocyte quantity rather than oocyte quality (32). If the primordial follicles that survive surgical damage retain normal developmental competence, the resulting embryos—though fewer in number—may have comparable implantation potential per transfer, consistent with our observation that per-transfer pregnancy and live birth rates did not differ significantly between groups. Third, our multivariable analysis identified patient age (OR = 0.92 per year, P = 0.030) and number of transferred embryos (OR = 2.21, P = 0.014) as the only independent predictors of CLBR, while surgical status was not a significant predictor. This finding suggests that the biological determinants of cumulative reproductive success are dominated by maternal age-related oocyte competence and the opportunity for multiple embryo transfers, rather than by the absolute number of oocytes retrieved in a single cycle. Fourth, the potential improvement in the pelvic microenvironment following cystectomy—including reduction in inflammatory cytokine exposure and resolution of peri-ovarian adhesions—may partially offset the quantitative reduction in gamete yield, although this hypothesis requires validation with prospective follicular fluid analyses (33, 34).

Empirical support for the cumulative FET buffer hypothesis can be drawn from the present data: the non-significant difference in cumulative pregnancy rates between Groups B and C (70.21% vs. 60.61%, P = 0.222) despite significantly fewer embryos in Group C directly demonstrate this compensatory mechanism in action. Furthermore, the finding that number of transferred embryos—rather than number of retrieved oocytes—was an independent predictor of CLBR (OR = 2.21, P = 0.014) substantiates the primacy of transfer opportunity over retrieval efficiency in determining cumulative reproductive success.

Our findings are consistent with prior systematic reviews reporting similar IVF outcomes between surgically and non-surgically managed OMA patients (4, 34). Recent multicenter retrospective analyses have similarly demonstrated that prior cystectomy did not improve live birth rates per started cycle while significantly reducing ovarian response (35, 36). Updated ESHRE guidelines (2022) now acknowledge the growing evidence supporting expectant management or direct IVF when fertility is the primary concern, particularly in patients with diminished ovarian reserve (1). The ASRM Practice Committee similarly cautions that the decision to perform surgery should carefully weigh the risk of reducing ovarian reserve against any potential benefit (37).

These converging lines of evidence challenge the routine recommendation for pre-IVF cystectomy in OMA ≥4 cm patients and support direct IVF as a reasonable first-line strategy for patients without absolute surgical indications.

Most notably, a 2025 systematic review and meta-analysis of 22 studies encompassing 3,590 participants directly compared surgery-first versus direct IVF/ICSI in women with OMA and deep infiltrating endometriosis (38). Surgery followed by IVF/ICSI did not significantly improve live birth rate in patients with OMA (OR = 0.89, 95% CI: 0.68–1.16), while oocyte retrieval was significantly lower in the surgery group. These pooled findings, derived from the largest and most methodologically rigorous synthesis to date, provide strong corroborative evidence for our observation that pre-IVF cystectomy does not improve cumulative reproductive outcomes.

Our findings acquire additional significance in the context of a recent large retrospective cohort study by Li et al. (2026), which evaluated the impact of endometriotic cystectomy on ovarian reserve and ovulation induction outcomes in 833 patients across three groups (non-endometriosis controls, non-surgical endometrioma, and post-cystectomy) at a Chinese IVF center during a comparable study period (2016–2022) (19). Li et al. confirmed that prior cystectomy was associated with significantly lower AMH levels, reduced AFC, higher FSH, and fewer retrieved oocytes compared with both non-surgical endometriosis patients and non-endometriosis controls—findings that are entirely consistent with the ovarian reserve impairment documented in our study. However, Li et al. did not collect pregnancy or live birth data, which the authors explicitly acknowledged as a major limitation. Consequently, whether the well-documented surgical damage to ovarian reserve translates into impaired cumulative reproductive outcomes remained unanswered. The present study directly addresses this critical evidence gap by demonstrating that, despite the reduction in oocyte yield and embryological efficiency following cystectomy, cumulative live birth rate was not significantly compromised—a finding that remained robust across multivariable regression (adjusted OR = 0.77, P = 0.469), propensity score matching (OR = 0.67, P = 0.374), and inverse probability of treatment weighting (OR = 0.77, P = 0.448). Furthermore, our study extends the existing evidence base in several additional dimensions not addressed by Li et al.: stratification by OMA size using the clinically relevant 4 cm threshold recommended by current guidelines, comprehensive embryological data spanning the full cascade from oocyte retrieval to blastocyst formation, and exploratory subgroup analyses across age, AMH level, embryo type, and OMA laterality. Collectively, our findings complement and extend those of Li et al. by completing the translational bridge from surrogate markers of ovarian damage to the patient-centered outcome that ultimately matters most—the probability of achieving a live birth.

### 4.3 Surgical damage to ovarian reserve: implications for fertility preservation

The present study provides direct evidence that laparoscopic cystectomy is associated with substantial and clinically meaningful damage to ovarian reserve. Patients in the surgical group had significantly lower serum AMH (1.98 vs. 3.16 ng/mL, representing a 37% reduction), fewer retrieved oocytes (median 7 vs. 11, a 36% reduction), and impaired embryological outcomes including lower 2PN rate, cleavage rate, and blastocyst formation rate. Multiple linear regression confirmed that prior cystectomy was independently associated with retrieval of approximately 2 fewer oocytes per cycle (β=−1.92, P = 0.015), independent of baseline ovarian reserve parameters.

These findings are consistent with a substantial body of literature documenting postoperative AMH decline. Multiple systematic reviews have demonstrated that serum AMH decreases by approximately 39% following unilateral and 57% following bilateral OMA excision (15, 39). Histopathological studies consistently document inadvertent loss of primordial follicles in the ovarian cortex adjacent to the excised cyst wall (40). The mechanisms of surgical harm include thermal injury from electrocautery hemostasis, inadvertent stripping of functional cortical tissue, and disruption of ovarian vascularity.

The choice of hemostatic technique following stripping cystectomy is increasingly recognized as a critical determinant of postoperative ovarian reserve decline. A network meta-analysis of randomized controlled trials by Riemma et al. (2023) demonstrated that bipolar electrocoagulation results in significantly greater AMH decline compared with suture-based hemostasis and hemostatic sealants (41). More recently, Aslan et al. (2025) reported prospective longitudinal data showing that energy-based hemostatic modalities cause the most pronounced decrease in AMH levels, whereas suturing and newer ovary-sparing techniques may partially mitigate this damage (42). These findings underscore that the surgical damage documented in our study likely encompasses a heterogeneous spectrum of ovarian injury dependent on the specific hemostatic approach employed—information that was unavailable for the majority of Group C patients who underwent cystectomy at external institutions.

Given the irreversible nature of surgical ovarian damage, preoperative fertility preservation (FP) merits serious consideration for patients with OMA ≥4 cm in whom surgery is planned. This approach involves cryopreserving oocytes or embryos prior to surgical intervention, thereby securing a reproductive “safety net.” Cobo et al. demonstrated that OMA patients undergoing pre-surgical FP retrieve significantly more oocytes than those proceeding directly to cystectomy (43). The 2022 Chinese Expert Consensus explicitly endorses pre-surgical FP in patients with diminished ovarian reserve (44), and we advocate extending this recommendation to include patients with normal preoperative reserve in whom significant postoperative decline is anticipated.

### 4.4 Safety considerations for direct IVF with OMA *in situ*

A clinical strategy of proceeding directly to IVF without prior cystectomy requires careful consideration of the safety implications of performing oocyte retrieval in the presence of an intact OMA. Three principal concerns have been raised in the literature: inadvertent puncture of the endometrioma during transvaginal oocyte retrieval, subsequent pelvic infection, and the theoretical impact of endometriotic fluid contamination on oocyte or embryo quality.

Regarding the risk of pelvic infection, the available evidence is reassuring. Benaglia et al. (2014) reported that the incidence of pelvic abscess following oocyte retrieval in patients with OMA was exceedingly low, comparable to that observed in women without endometriomas, provided that standard antibiotic prophylaxis and aseptic technique were employed (45). A comprehensive review by Somigliana et al. (2015) similarly concluded that the presence of OMA does not substantially increase the risk of serious infectious complications during IVF procedures, and that the absolute risk of clinically significant pelvic abscess remains very rare across published series (46).

Concerns regarding contamination of retrieved oocytes by endometriotic cyst fluid have also been investigated. While some early reports suggested that inadvertent aspiration of endometrioma contents could expose follicular fluid to inflammatory cytokines and oxidative stress mediators, subsequent studies have demonstrated that careful ultrasound-guided needle placement can avoid cyst puncture in the majority of cases (47). When accidental puncture does occur, the clinical impact on embryo quality and pregnancy outcomes appears to be minimal, although operators should exercise caution to minimize this risk by selecting a needle trajectory that avoids the endometrioma whenever anatomically feasible.

A further practical concern is that large OMAs may physically obstruct access to adjacent follicles, potentially reducing the number of retrievable oocytes. However, this mechanical effect should be distinguished from genuine ovarian damage: it represents a procedural limitation of a single retrieval cycle rather than an irreversible reduction in reproductive potential. In the present study, patients with non-surgically managed OMA ≥4 cm (Group B) achieved a median of 11 retrieved oocytes—a yield that was not significantly different from that of patients with smaller cysts (Group A) and substantially higher than that of post-surgical patients (Group C, median 7)—suggesting that the mechanical impact of intact OMA on oocyte retrieval efficiency was limited.

Taken together, the current evidence indicates that direct IVF in the presence of OMA is a safe and feasible approach when performed by experienced operators with appropriate precautions. The low complication rates reported in the literature, combined with the preservation of ovarian reserve demonstrated in our study, support the consideration of direct IVF as a reasonable first-line strategy for infertile patients with OMA ≥4 cm in whom there is no absolute surgical indication.

### 4.5 Clinical implications and individualized management framework

Based on the findings of this study, interpreted in the context of existing literature, we propose the following preliminary clinical framework for individualized management of infertile patients with OMA. This framework is intended to inform shared decision-making and requires validation in prospective multicenter studies:

1. OMA <4 cm with infertility: Direct IVF without prior surgical intervention appears to be a reasonable approach. Our data suggest that cyst size within this range does not independently influence oocyte yield, embryo quality, or cumulative live birth rate, although this comparison was underpowered (5.1% power) and requires confirmation in larger cohorts.
2. OMA ≥4 cm with infertility and no absolute surgical indication: Direct IVF may be considered as a reasonable first-line strategy, particularly in women of advancing reproductive age in whom surgical delay could compromise time-sensitive fertility potential. If IVF is successful, surgical intervention can be deferred or reconsidered based on symptom burden and long-term management goals. This approach should be weighed against the low but non-negligible risks of OMA malignant transformation (estimated 0.3–1.0% lifetime risk) and the theoretical risks associated with oocyte retrieval in the presence of an intact endometrioma, which appear to be very low based on available evidence (46–48). Individualized counseling incorporating patient preferences, symptom severity, cyst characteristics, and ovarian reserve status is essential.

It should be noted that the management of OMA before IVF is not limited to the binary choice between laparoscopic cystectomy and expectant management. Ultrasound-guided aspiration of endometrioma contents, with or without ethanol sclerotherapy, has been proposed as a minimally invasive alternative that may reduce cyst size and improve follicular accessibility without the tissue destruction inherent to surgical excision (49, 50). Some centers have adopted the practice of aspirating endometriomas at the time of oocyte retrieval, thereby combining diagnostic and therapeutic steps in a single procedure (51). However, evidence supporting this approach remains limited: recurrence rates following simple aspiration are high (ranging from 30% to 80%), long-term reproductive outcomes are poorly characterized, and no randomized trials have directly compared aspiration-based strategies with either cystectomy or direct IVF using cumulative live birth rate as the primary endpoint (52). Although aspiration-based approaches were not evaluated in the present study, they represent a potentially important “middle-ground” strategy that warrants investigation in future prospective trials, particularly for patients with large OMAs who wish to avoid both formal surgery and the theoretical risks of leaving a large cyst untreated during IVF.

(3) OMA ≥4 cm with infertility and planned surgery (for symptom management, diagnostic purposes, or other clinical indications): Preoperative fertility preservation through oocyte or embryo cryopreservation should be discussed with the patient prior to cystectomy, given the well-documented risk of post-surgical ovarian reserve decline (15, 39). This recommendation is particularly pertinent for patients with already diminished ovarian reserve or those with bilateral endometriomas, in whom the anticipated post-surgical decline may be most consequential.

Patient age emerged as the most robust independent predictor of cumulative live birth rate in our analysis (OR = 0.92 per year, P = 0.030), consistent with the well-established primacy of maternal age in determining IVF success. This finding carries important implications for treatment sequencing: time spent pursuing surgical intervention—with associated recovery periods and the potential need for repeat procedures—may itself compromise reproductive success by advancing maternal age during a period of accelerating fertility decline. Clinicians should therefore carefully balance the potential benefits of surgery against the biological cost of treatment delay, particularly in women aged 35 years and older.

The temporal and economic costs of pre-IVF surgical intervention merit explicit consideration in clinical decision-making. The pathway from surgical consultation to post-operative recovery and subsequent initiation of IVF typically spans 3 to 6 months, encompassing pre-operative evaluation, surgical scheduling, the procedure itself, and a recommended recovery interval of at least 2–3 months to allow ovarian function to stabilize before commencing controlled ovarian stimulation (53). In some clinical settings, this timeline may be further prolonged by surgical waiting lists, post-operative complications, or the need for adjunctive GnRH agonist therapy. For women of advancing reproductive age, this delay carries a quantifiable biological cost: live birth rates decline progressively after age 35, with ART registry and population-based studies consistently showing worse outcomes at older ages (54, 55)., and our own data confirm that age was the strongest independent predictor of CLBR (OR = 0.92 per year, P = 0.030). Consequently, a 6-month surgical delay in a 36-year-old patient represents not merely a logistical inconvenience but a measurable reduction in reproductive potential that may not be recovered.

From an economic perspective, the surgical pathway incurs substantial direct costs-including operating room fees, anesthesia, hospitalization, pathology, and post-operative follow-up-in addition to indirect costs such as lost productivity and time away from work (56). These expenditures are incurred before the patient even begins IVF, with no guarantee of improved reproductive outcomes, as demonstrated by our findings. By contrast, direct IVF consolidates the treatment pathway, potentially reducing both the time to pregnancy and the overall financial burden. While formal cost-effectiveness analyses comparing surgical versus non-surgical management of OMA prior to IVF are currently lacking, the convergence of comparable CLBR, reduced time to treatment, and avoidance of surgical costs suggests that direct IVF may offer a more efficient allocation of both biological and economic resources. Future studies incorporating health-economic modeling would be valuable to quantify these potential advantages.

### 4.6 Methodological considerations

The marked imbalance in group sizes between non-surgical (Group B, n=47) and surgical (Group C, n=231) OMA ≥4 cm cohorts warrants discussion. This distribution reflects prevailing clinical practice where laparoscopic cystectomy has been the standard recommendation for OMA ≥4 cm per national guidelines, and paradoxically underscores the clinical relevance of our research question—the very guideline we are evaluating has limited the pool of non-surgically managed patients available for study.

Several design features strengthen confidence in our findings despite this imbalance. First, propensity score matching successfully identified 41 matched pairs with improved covariate balance, and results remained consistent (OR = 0.67, P = 0.374). Second, IPTW analysis of the full cohort yielded concordant results (OR = 0.77, P = 0.448). Third, exploratory subgroup analyses revealed consistent treatment effects across age, AMH, embryo type, and laterality subgroups, with no significant interaction effects. The convergence of these three analytical approaches—multivariable regression, PSM, and IPTW-strengthens the inference that surgical status does not independently predict cumulative live birth rate.

The single-center design, while limiting generalizability, confers the advantage of standardized diagnostic criteria, surgical techniques (for external referrals notwithstanding), and IVF protocols, thereby reducing heterogeneity that might confound multicenter studies.

We acknowledge that the non-significant findings should be interpreted with caution given the limited statistical power of this comparison. The absolute difference in CLBR between Groups B and C (59.6% vs. 52.4%, Δ=7.2 percentage points) is clinically non-trivial, and the 95% confidence interval for this absolute difference (−22.6% to +8.2%) is wide enough to encompass both clinical equivalence and a substantial detriment of cystectomy. The PSM-adjusted OR of 0.67—while not statistically significant (P = 0.374)—is consistent with a trend toward lower CLBR in the surgical group. Given the small sample size of Group B (n=47), a false-negative finding due to insufficient power cannot be excluded; the minimum detectable difference with 80% power was 22.4 percentage points, far exceeding the observed 7.2-point difference. Our results should therefore be interpreted as absence of evidence for a large detrimental effect of surgery on CLBR, rather than definitive evidence of equivalence.

It is important to emphasize that the present study was designed as a superiority analysis, not a non-inferiority or equivalence trial. The failure to detect a statistically significant difference in CLBR between surgical and non-surgical management does not constitute evidence that the two strategies produce equivalent reproductive outcomes. The confidence interval for the primary comparison (adjusted OR: 0.38–1.56) spans a range from a 62% reduction to a 56% increase in the odds of live birth with cystectomy—a range far too wide to support any claim of equivalence. Establishing true non-inferiority would require a prospective trial with a pre-specified non-inferiority margin, adequate sample size calculated for one-sided testing, and an intention-to-treat analysis framework—none of which are features of the current retrospective design. Accordingly, our findings should be interpreted strictly as failure to demonstrate a statistically significant benefit of cystectomy within the limitations of the available sample, rather than as evidence supporting the interchangeability of surgical and non-surgical management.

### 4.7 Limitations and future directions

Several limitations of this study warrant acknowledgment and should inform the interpretation of our findings.

First, the retrospective design precludes random allocation and cannot fully exclude residual confounding or selection bias. Patients in the non-surgical OMA ≥4 cm group (Group B) may represent a selected population: those who declined surgery or were counseled against it may have had more favorable baseline characteristics—such as higher ovarian reserve, fewer symptoms, or smaller cyst burden—that independently predispose to better IVF outcomes. Although we employed multivariable regression, propensity score matching, and IPTW to mitigate measured confounding, unmeasured confounders may persist. E-value analysis revealed that relatively modest unmeasured confounding (E-value = 1.92 for the point estimate of the surgery comparison) could account for the observed association, and since the confidence interval already crossed the null (E-value for CI limit = 1.0), our findings cannot definitively exclude clinically meaningful effects of surgical management on CLBR. This underscores the need for adequately powered randomized trials to provide definitive evidence.

Furthermore, an inherent diagnostic asymmetry exists between the study groups: non-surgical patients were diagnosed by imaging criteria alone, whereas surgical patients had pathological confirmation of endometrioma. Although transvaginal ultrasound demonstrates high diagnostic accuracy for OMA (sensitivity 93%, specificity 96%) (23), the possibility of diagnostic misclassification in the non-surgical group cannot be excluded. Additionally, the criteria underlying surgical decision-making—including symptom severity, cyst characteristics, patient preference, and physician judgment—were not systematically captured in this retrospective design. It is plausible that physicians selectively recommended surgery for patients perceived to have poorer prognoses, introducing indication bias that PSM and IPTW can only partially address when limited to measured covariates.

Second, the non-surgical OMA ≥4 cm group (Group B, n = 47) was relatively small, reflecting current clinical practice where most patients with large OMA undergo surgery per guideline recommendations. The 95% confidence interval for the absolute CLBR difference between Groups A and B ranged from −15.7% to +17.6%, and between Groups B and C from −22.6% to +8.2%, indicating that neither comparison can exclude clinically meaningful differences. The minimum detectable difference with 80% power was 23.8 and 22.4 percentage points respectively, confirming that both comparisons were substantially underpowered. These findings should therefore be interpreted as absence of evidence for a large effect rather than definitive evidence of equivalence or non-inferiority.

Third, the interval between laparoscopic cystectomy and initiation of IVF was not available for analysis in Group C patients. This surgery-to-IVF interval is a potentially important variable: shorter intervals may reflect insufficient time for ovarian recovery, while longer intervals may introduce additional age-related fertility decline. Previous studies have suggested that AMH levels may partially recover within 3–6 months following cystectomy (57), and the timing of IVF initiation relative to surgery could modulate the observed effect on oocyte yield and embryological outcomes. The inability to account for this variable represents an important limitation that may have introduced unquantified confounding in either direction.

Fourth, complete operative records—including detailed documentation of surgical technique, hemostatic method (electrocautery versus suturing), extent of ovarian tissue excision, and standardized endometriosis staging (rASRM classification)—were unavailable for many patients in Group C, as a substantial proportion had undergone cystectomy at external institutions prior to referral to our center. Surgical technique is a recognized determinant of post-operative ovarian reserve decline: bipolar electrocoagulation for hemostasis has been associated with greater thermal damage to ovarian cortex compared with suture-based techniques, and surgeon experience may influence the extent of inadvertent removal of healthy ovarian tissue (58–61). The heterogeneity in surgical approach within Group C—which could not be characterized or adjusted for in our analyses—limits our ability to determine whether the observed surgical damage reflects an inherent consequence of cystectomy or is partly attributable to variations in surgical quality. Future studies should incorporate standardized documentation of surgical technique and endometriosis staging to enable more precise assessment of the relationship between surgery and IVF outcomes.

In light of recent evidence demonstrating significant differences in ovarian reserve outcomes between hemostatic techniques (41, 42), this limitation is particularly consequential: the surgical group may include patients who experienced varying degrees of iatrogenic ovarian damage depending on the hemostatic method used, and this uncharacterized heterogeneity may have either attenuated or amplified the observed differences in IVF outcomes.

Fifth, although our institutional data confirmed that the distribution of COS protocols (including the proportion of patients receiving GnRH agonist ultra-long down-regulation) was comparable across study groups at baseline, detailed protocol-specific data were not available for inclusion in the present analysis. Differences in ovarian stimulation strategies could theoretically influence oocyte yield and embryological outcomes independently of OMA management, and future studies should systematically report and adjust for COS protocol as a potential confounder.

Sixth, the single-center design, while conferring the advantage of standardized IVF laboratory protocols and consistent clinical practice, limits the generalizability of our findings to other clinical settings with different patient populations, surgical expertise, or IVF protocols.

The exclusion of patients with concomitant adenomyosis, while methodologically justified to isolate the effect of OMA management on IVF outcomes, limits the generalizability of our findings to the substantial proportion of endometriosis patients with coexisting adenomyosis. Given recent evidence that adenomyosis independently impairs implantation and obstetric outcomes (25, 26), future studies should specifically examine how concomitant adenomyosis modifies the relationship between OMA management strategy and CLBR.

The exclusion of patients with endocrine comorbidities—including thyroid dysfunction, diabetes mellitus, and polycystic ovary syndrome—was implemented to reduce confounding, but this design choice limits the applicability of our findings to the broader clinical population of endometriosis patients, among whom these comorbidities are prevalent. Given the close relationship between endometriosis and endocrine dysregulation, future studies incorporating these populations would provide more generalizable estimates.

Future research should prioritize prospective, multicenter randomized controlled trials comparing pre-IVF cystectomy with direct IVF—with or without preoperative fertility preservation—in patients with OMA ≥4 cm, using cumulative live birth rate as the primary endpoint. Such trials should incorporate standardized endometriosis staging, detailed documentation of surgical technique, stratification by surgery-to-IVF interval, formal cost-effectiveness analysis, and patient-reported outcomes including quality of life and treatment satisfaction, to provide the comprehensive evidence base needed to refine clinical guidelines and optimize individualized management strategies. Specifically, such a trial would ideally adopt a non-inferiority design with a pre-specified margin (e.g., 10 percentage points in absolute CLBR difference), one-sided α=0.025, and a sample size of approximately 744 patients per group to provide 80% power to detect the 7.2-percentage-point difference observed in the present study. An intention-to-treat analysis framework with stratification by age, AMH level, and OMA laterality would further strengthen causal inference.

The current study design cannot fully disentangle the effect of cystectomy on ovarian reserve from its potential effects on the uterine and pelvic environment. Cystectomy may simultaneously reduce oocyte yield through ovarian damage while improving implantation conditions through reduction of pelvic inflammation and adhesions. A design that separates these effects—for example, oocyte retrieval and embryo cryopreservation before surgery, followed by frozen embryo transfer after surgical recovery—would more precisely delineate the independent contributions of ovarian versus uterine factors to reproductive outcomes.

## 5 Conclusions

This retrospective cohort study of 394 infertile patients with ovarian endometrioma did not detect a statistically significant benefit of pre-IVF laparoscopic cystectomy on cumulative live birth rate in patients with OMA ≥4 cm; however, the wide confidence intervals preclude definitive conclusions regarding equivalence between surgical and non-surgical management.

In non-surgically managed patients, OMA size (<4 cm vs. ≥4 cm) did not independently predict oocyte yield, embryological outcomes, or cumulative live birth rate, although this comparison was underpowered and requires confirmation in larger studies. Among patients with OMA ≥4 cm, prior cystectomy was associated with significantly reduced ovarian reserve (AMH reduction of 37%), fewer retrieved oocytes (median 7 vs. 11), and impaired embryological efficiency—yet the difference in cumulative live birth rates between surgical and non-surgical management did not reach statistical significance (52.4% vs. 59.6%; adjusted OR = 0.77, 95% CI: 0.38–1.56, P = 0.469). These findings were robust across propensity score matching (OR = 0.67, P = 0.374) and inverse probability of treatment weighting analyses (OR = 0.77, P = 0.448).

Patient age (OR = 0.92 per year, P = 0.030) and number of transferred embryos (OR = 2.21 for 2 vs. 1, P = 0.014)—rather than OMA size or surgical status—were the only independent predictors of cumulative live birth rate, underscoring the primacy of time-sensitive factors in determining reproductive success.

These findings generate the following hypotheses for prospective validation (1): direct IVF without prior cystectomy may be considered as an alternative strategy worthy of further investigation for infertile patients with OMA ≥4 cm in whom there is no absolute surgical indication (2); for patients in whom surgery is planned for other clinical indications, preoperative fertility preservation through oocyte or embryo cryopreservation should be discussed to safeguard reproductive potential; and (3) treatment delays associated with surgical intervention should be carefully weighed against the biological cost of advancing maternal age.

Given the inherent limitations of retrospective single-center evidence—including potential indication bias, limited statistical power, and the inability to establish causality—these findings should be regarded as hypothesis-generating. Definitive clinical recommendations await adequately powered, prospective, multicenter randomized controlled trials using CLBR as the primary endpoint. These findings neither establish that direct IVF is non-inferior to cystectomy nor confirm that cystectomy provides a reproductive benefit, and should not be used as sole justification for changes in clinical practice or guideline recommendations.

## Data Availability

The raw data supporting the conclusions of this article will be made available by the authors, without undue reservation.

## Supplementary material

Supplementary Figure 1 Forest plot of exploratory subgroup analyses comparing cumulative live birth rate between Group B (non-surgical OMA ≥4 cm, n = 47) and Group C (surgical OMA ≥4 cm, n = 231). The forest plot displays odds ratios (OR) with 95% confidence intervals (CI) for the effect of surgical versus non-surgical management on cumulative live birth rate across pre-specified subgroups. The vertical dashed line represents OR = 1 (no difference). An OR < 1 favors the surgical group, while an OR > 1 favors the non-surgical group. The overall effect is shown as an orange square; subgroup estimates are shown as blue diamonds. Subgroups examined include: (1) age (<35 vs. ≥35 years); (2) ovarian reserve status based on anti-Müllerian hormone level (AMH ≥1.1 vs. <1.1 ng/mL); (3) embryo type at transfer (cleavage-stage vs. blastocyst); and (4) OMA laterality (unilateral vs. bilateral). All P-interaction values exceeded 0.05, indicating no significant effect modification. AMH, anti-Müllerian hormone; CI, confidence interval; OMA, ovarian endometrioma; OR, odds ratio; P-int, P-value for interaction.
