# Supplementary material for: Direct IVF versus prior laparoscopic cystectomy for ovarian endometrioma ≥4 cm: cumulative live birth rate and implications for fertility preservation — a retrospective cohort study
Source: Front Endocrinol (Lausanne). 2026 Jul 31;17:1851907. doi: 10.3389/fendo.2026.1851907 (PMC13472831; doi:10.3389/fendo.2026.1851907)
Supplement: Supplementary file 1 [file Table1.docx]

**Table S1. Controlled ovarian stimulation parameters and embryological outcomes in patients with ovarian endometrioma managed non-surgically: OMA <4 cm (Group A) versus OMA ≥4 cm (Group B)**

| **Variable** | **Group A (n=116)**  **Non-surgical OMA <4 cm** | **Group B (n=47)**  **Non-surgical OMA ≥4 cm** | **Z/χ² value** | **P value** |
| --- | --- | --- | --- | --- |
| ***Controlled Ovarian Stimulation Parameters*** | | | | |
| Starting Gn dose (IU) | 150.00 (125.00, 225.00) | 162.50 (125.00, 225.00) | 0.574 | 0.569 |
| Total Gn dose (IU) | 2175.00 (1743.75, 3093.75) | 2325.00 (1800.00, 2775.00) | −0.126 | 0.908 |
| Duration of Gn stimulation (days) | 11.00 (10.00, 12.25) | 11.00 (10.00, 12.00) | −0.707 | 0.484 |
| E2 on trigger day (pg/mL) | 1883.50 (976.78, 2934.25) | 1955.50 (1057.75, 3000.00) | −0.487 | 0.635 |
| LH on trigger day (IU/L) | 0.95 (0.49, 2.62) | 1.12 (0.68, 2.07) | −0.317 | 0.765 |
| P on trigger day (ng/mL) | 0.70 (0.46, 0.96) | 0.69 (0.41, 1.03) | −0.194 | 0.853 |
| Retrieved oocytes (count) | 10.00 (6.00, 15.00) | 11.00 (6.00, 15.00) | −0.168 | 0.874 |
| MII oocytes (count) | 8.00 (5.00, 12.00) | 9.00 (6.00, 12.50) | −0.656 | 0.515 |
| MII oocyte rate (%) | 83.64% (1048/1253) | 87.43% (445/509) | 4.015 | **0.045*** |
| ***Embryological Outcomes*** | | | | |
| Fertilization method |  |  |  | 0.413 |
| Conventional IVF | 81.90% (95/116) | 87.23% (41/47) |  |  |
| ICSI | 18.10% (21/116) | 12.77% (6/47) |  |  |
| Fertilization rate (%) | 72.04% (964/1253) | 80.94% (412/509) | 3.404 | 0.078 |
| 2PN count (per patient) | 6.00 (3.00, 9.00) | 6.00 (3.50, 10.00) | −1.176 | 0.240 |
| 2PN rate (%) | 72.04% (755/1048) | 77.98% (347/445) | 5.697 | **0.020*** |
| Cleavage rate (%) | 84.96% (819/964) | 86.65% (357/412) | 0.677 | 0.426 |
| Available Day-3 embryos (count) | 4.00 (3.00, 7.00) | 5.00 (3.00, 8.00) | 1.407 | 0.168 |
| Top-quality embryos (count) | 0.00 (0.00, 2.00) | 2.00 (0.00, 3.50) | −1.444 | 0.156 |
| Blastocyst count (per patient) | 1.00 (0.00, 4.00) | 2.00 (0.00, 4.00) | −0.826 | 0.414 |
| Blastocyst formation rate (%) | 22.50 (0.00, 69.81) | 50.00 (0.00, 69.05) | −1.049 | 0.308 |

**Notes:** Data are presented as median (interquartile range) for continuous variables and % (n/N) for rates. Statistical comparisons were performed using the Mann-Whitney U test for continuous variables and the chi-square test for categorical variables. Group A: non-surgical management of OMA <4 cm; Group B: non-surgical management of OMA ≥4 cm. *P < 0.05.

**Abbreviations:** 2PN, two pronuclei; E2, estradiol; Gn, gonadotropin; ICSI, intracytoplasmic sperm injection; IVF, in vitro fertilization; LH, luteinizing hormone; MII, metaphase II; OMA, ovarian endometrioma; P, progesterone.

**Table S2. Clinical Pregnancy Outcomes: Group A vs Group B**

| **Variable** | **Group A (n=116)** | **Group B (n=47)** | **Z/x² value** | **P value** |
| --- | --- | --- | --- | --- |
| Endometrial thickness at transfer (mm) | 11.00 (9.50, 13.00) | 10.00 (9.00, 12.00) | -1.303 | 0.203 |
| First transfer cycle type (%) | | | 0.750 | 0.393 |
| Fresh cycle | 54.31% (63/116) | 46.81% (22/47) | |  |
| Frozen-thawed cycle | 45.69% (53/116) | 53.19% (25/47) | |  |
| Number of transferred embryos (%) | | | 1.325 | 0.250 |
| 1 embryo | 37.07% (43/116) | 46.81% (22/47) | |  |
| 2 embryos | 62.93% (73/116) | 53.19% (25/47) | |  |
| Type of transferred embryos (%) | | | 1.375 | 0.242 |
| Cleavage-stage | 75.00% (87/116) | 65.96% (31/47) | |  |
| Blastocyst | 25.00% (29/116) | 34.04% (16/47) | |  |
| Embryo implantation rate (%) | 47.06% (88/187) | 54.17% (39/72) | 0.369 | 0.558 |
| Fresh cycle clinical pregnancy rate (%) | 58.73% (37/63) | 68.18% (15/22) | 0.618 | 0.436 |
| Fresh cycle live birth rate (%) | 55.56% (35/63) | 54.55% (12/22) | 0.017 | 0.935 |
| FET clinical pregnancy rate (%) | 67.92% (36/53) | 72.00% (18/25) | 0.136 | 0.724 |
| FET live birth rate (%) | 62.26% (33/53) | 64.00% (16/25) | 0.025 | 0.884 |
| Ectopic pregnancy rate (%) | 0.00% (0/116) | 2.13% (1/47) | 2.484 | 0.123 |
| Miscarriage rate (%) | 4.31% (5/116) | 8.51% (4/47) | 4.000 | 0.261 |
| Cumulative pregnancy rate (%) | 62.93% (73/116) | 70.21% (33/47) | 0.783 | 0.380 |
| Cumulative live birth rate (%) | 58.62% (68/116) | 59.57% (28/47) | 0.011 | 0.911 |

**Notes**: Data are presented as n (%). Clinical pregnancy was defined as the presence of an intrauterine gestational sac with fetal cardiac activity on transvaginal ultrasound at 6-7 weeks of gestation. Early miscarriage was defined as pregnancy loss before 12 weeks of gestation. Cumulative outcomes include all fresh and frozen embryo transfer cycles until first live birth or exhaustion of all available embryos within the study period.

**Abbreviations**: CI, confidence interval; FET, frozen embryo transfer; IQR, interquartile range.

**Table S3. Multiple Linear Regression: Determinants of Retrieved Oocyte Number (Group A vs Group B)**

| **Variable** | **β** | **S.E.** | **t** | **P value** | **β (95% CI)** |
| --- | --- | --- | --- | --- | --- |
| Group (reference: Group A) | | |  |  | 1 |
| Group B | 0.29 | 1.07 | 0.27 | 0.954 | 0.29 (-2.00 to 1.89) |
| Age | -0.49 | 0.15 | -3.16 | **0.002*** | -0.49 (-0.79 to -0.19) |
| BMI | -0.09 | 0.15 | -0.63 | 0.529 | -0.09 (-0.38 to 0.19) |
| AMH | 0.21 | 0.17 | 1.24 | 0.218 | 0.21 (-0.12 to 0.53) |
| Basal AFC | 0.16 | 0.12 | 1.24 | 0.216 | 0.16 (-0.09 to 0.40) |
| Duration of infertility | -0.07 | 0.21 | -0.32 | 0.750 | -0.07 (-0.48 to 0.35) |
| Basal FSH | -0.31 | 0.2 | -1.52 | 0.131 | -0.31 (-0.71 to 0.09) |
| Starting Gn dose | 0 | 0.01 | 0.14 | 0.887 | 0.00 (-0.02 to 0.02) |
| Total Gn dose | 0 | 0 | -0.33 | 0.742 | -0.00 (-0.00 to 0.00) |
| COS protocol (reference: ultra-long) | | | |  | 1 |
| Long protocol | 1.89 | 2.94 | 0.64 | 0.522 | 1.89 (-3.88 to 7.66) |
| Antagonist protocol | -1.21 | 3.45 | -0.35 | 0.727 | -1.21 (-7.97 to 5.55) |
| Non-downregulation protocol | 0.84 | 3.9 | 0.22 | 0.830 | 0.84 (-6.81 to 8.49) |

**Notes**: Multiple linear regression analysis with retrieved oocyte number as the dependent variable. The model was adjusted for potential confounders including age, BMI, AMH, AFC, and infertility duration. Standardized coefficients (β) represent the change in standard deviation units of the dependent variable per one standard deviation change in the independent variable. Model assumptions (linearity, homoscedasticity, normality of residuals, and absence of multicollinearity) were verified. Variance inflation factors (VIF) were all <2.0, indicating no significant multicollinearity.

**Abbreviations**: AFC, antral follicle count; AMH, anti-Müllerian hormone; BMI, body mass index; CI, confidence interval; OMA, ovarian endometrioma; SE, standard error; VIF, variance inflation factor.

**Table S4. Multiple Linear Regression: Determinants of Retrieved Oocyte Number (Group B vs Group C)**

| **Variable** | **β** | **SE** | **t** | **P value** | **β (95% CI)** |
| --- | --- | --- | --- | --- | --- |
| Group (reference: Group B) | | |  |  |  |
| Group C | -1.92 | 0.79 | -2.43 | **0.015*** | -1.92 (-3.48 to -0.39) |
| Age | -0.12 | 0.09 | -1.34 | 0.180 | -0.12 (-0.31 to 0.06) |
| BMI | -0.20 | 0.10 | -2.06 | **0.040*** | -0.20 (-0.40 to -0.01) |
| AMH | 0.20 | 0.11 | 1.77 | 0.078 | 0.20 (-0.02 to 0.42) |
| Basal AFC | 0.22 | 0.07 | 3.01 | **0.003*** | 0.22 (0.08 to 0.36) |
| Basal FSH | -0.29 | 0.09 | -3.11 | **0.002*** | -0.29 (-0.48 to -0.11) |
| Starting Gn dose | -0.01 | 0.01 | -0.96 | 0.340 | -0.01 (-0.02 to 0.01) |
| Total Gn dose | 0 | 0 | -0.48 | 0.632 | -0.00 (-0.00 to 0.00) |
| Duration of infertility | -0.15 | 0.12 | -1.27 | 0.206 | -0.15 (-0.39 to 0.08) |
| COS protocol  (reference: ultra-long) | | | |  |  |
| Long protocol | -1.18 | 1.89 | -0.62 | 0.533 | -1.18 (-4.89 to 2.53) |
| Antagonist protocol | -1.92 | 2.03 | -0.95 | 0.343 | -1.92 (-5.90 to 2.05) |
| Non-downregulation  protocol | -3.24 | 2.37 | -1.36 | 0.174 | -3.24 (-7.89 to 1.42) |

**Notes**: Multiple linear regression analysis with retrieved oocyte number as the dependent variable. The model was adjusted for potential confounders including age, BMI, AMH, AFC, infertility duration, and bilateral OMA status. Unstandardized coefficients (B) represent the expected change in retrieved oocyte number per one-unit change in the independent variable. Group comparison (B vs C) uses Group B (non-surgical) as the reference category; therefore, the coefficient represents the difference in Group C relative to Group B. Model assumptions were verified as described in Table 3.3.

**Abbreviations**: AFC, antral follicle count; AMH, anti-Müllerian hormone; BMI, body mass index; CI, confidence interval; OMA, ovarian endometrioma; SE, standard error.

**Table S5. Univariable Logistic Regression: Factors Associated with Cumulative Live Birth Rate (Group A vs Group B)**

| **Variable** | **β** | **OR (95% CI)** | **P value** |
| --- | --- | --- | --- |
| Group (reference: Group A) | | |  |
| Group B | 0.04 | 1.04 (0.52-2.07) | 0.911 |
| Age | -0.07 | 0.93 (0.85-1.02) | 0.118 |
| BMI | 0.08 | 1.08 (0.97-1.21) | 0.138 |
| Basal FSH | -0.12 | 0.89 (0.77-1.02) | 0.088 |
| Basal LH | -0.08 | 0.92 (0.80-1.07) | 0.293 |
| AMH | -0.03 | 0.97 (0.88-1.08) | 0.620 |
| Basal AFC | 0.01 | 1.01 (0.95-1.08) | 0.701 |
| Starting Gn dose | 0 | 1.00 (0.99-1.00) | 0.113 |
| Total Gn dose | 0 | 1.00 (1.00-1.00) | 0.485 |
| Fertilization method (reference: IVF) | | | |
| ICSI | 0.6 | 1.82 (0.75-4.44) | 0.189 |
| Retrieved oocytes | 0.06 | 1.06 (1.01-1.12) | **0.043*** |
| Top-quality embryos | 0.05 | 1.05 (0.93-1.18) | 0.440 |
| First transfer cycle type  (reference: fresh cycle) | | | |
| Frozen-thawed cycle | -0.2 | 0.82 (0.44-1.53) | 0.537 |
| Number of transferred embryos (reference: 1) | | | |
| 2 embryos | 0.56 | 1.75 (0.92-3.30) | 0.087 |
| Type of transferred embryos (reference: cleavage-stage) | | | |
| Blastocyst | 0.06 | 1.07 (0.53-2.14) | 0.860 |
| Endometrial thickness at transfer | 0.01 | 1.01 (0.89-1.14) | 0.870 |

**Notes**: Univariable logistic regression analysis with cumulative live birth (defined as delivery of a live infant after 24 weeks of gestation) as the dependent variable. Odds ratios (OR) >1 indicate increased odds of live birth. Variables with P < 0.10 in univariable analysis were considered for inclusion in the multivariable model. Continuous variables were assessed for linearity in the logit; non-linear relationships were addressed by categorization or transformation as appropriate.

**Abbreviations**: AFC, antral follicle count; AMH, anti-Müllerian hormone; BMI, body mass index; CI, confidence interval; OMA, ovarian endometrioma; OR, odds ratio.

**Table S6. Univariable Logistic Regression: Factors Associated with Cumulative Live Birth Rate (Group B vs Group C)**

| **Variable** | **β** | **OR (95% CI)** | **P value** |
| --- | --- | --- | --- |
| Group (reference: Group B) | | |  |
| Group C | -0.29 | 0.75 (0.39-1.41) | 0.368 |
| Age | -0.07 | 0.93 (0.87-0.99) | **0.031*** |
| BMI | -0.01 | 0.99 (0.92-1.07) | 0.828 |
| Basal FSH | -0.05 | 0.95 (0.88-1.02) | 0.176 |
| Basal LH | -0.05 | 0.95 (0.87-1.04) | 0.239 |
| AMH | 0.08 | 1.08 (0.98-1.19) | 0.131 |
| Basal AFC | 0.04 | 1.04 (0.99-1.09) | 0.106 |
| Starting Gn dose | -0.01 | 0.99 (0.99-0.99) | **0.005*** |
| Total Gn dose | 0 | 1.00 (1.00-1.00) | 0.859 |
| Fertilization method  (reference: IVF) | | | |
| ICSI | 0.55 | 1.74 (0.78-3.89) | 0.178 |
| Retrieved oocytes | 0.03 | 1.03 (0.99-1.08) | 0.160 |
| Top-quality embryos | 0.04 | 1.04 (0.93-1.16) | 0.484 |
| First transfer cycle type  (reference: fresh cycle) | | | |
| Frozen-thawed cycle | -0.06 | 0.94 (0.57-1.55) | 0.816 |
| Number of transferred embryos  (reference: 1) | | | |
| 2 embryos | 0.55 | 1.74 (1.07-2.80) | **0.024*** |
| Type of transferred embryos  (reference: cleavage-stage) | | | |
| Blastocyst | -0.10 | 0.91 (0.51-1.61) | 0.735 |
| Endometrial thickness at transfer | -0.01 | 0.99 (0.91-1.08) | 0.841 |

**Notes**: Univariable logistic regression analysis with cumulative live birth (defined as delivery of a live infant after 24 weeks of gestation) as the dependent variable. Odds ratios (OR) >1 indicate increased odds of live birth. Variables with P < 0.10 in univariable analysis were considered for inclusion in the multivariable model. Continuous variables were assessed for linearity in the logit.

**Abbreviations**: AFC, antral follicle count; AMH, anti-Müllerian hormone; BMI, body mass index; CI, confidence interval; OMA, ovarian endometrioma; OR, odds ratio.

**Table S7. Propensity Score Matching Analysis: Baseline Characteristics Before and After Matching (Group B vs. Group C)**

| **Variable** | **Before Matching** |  | **SMD** | **After Matching**  **(1:1)** |  | **SMD** | **IPTW SMD** |
| --- | --- | --- | --- | --- | --- | --- | --- |
|  | Group B (n=45) | Group C (n=221) |  | Group B (n=41) | Group C (n=41) |  |  |
| Age (years) | 31.0 (29.0, 33.0) | 31.0 (29.0, 33.0) | −0.063 | 31.0 (29.0, 33.0) | 31.0 (30.0, 35.0) | 0.217 | 0.071 |
| BMI (kg/m²) | 21.3 (20.1, 23.0) | 21.6 (20.0, 23.9) | 0.186 | 21.5 (20.1, 23.0) | 20.7 (19.1, 23.3) | −0.258 | 0.112 |
| AMH (ng/mL) | 3.16 (1.97, 4.61) | 1.98 (1.18, 3.35) | −0.291 | 3.05 (1.90, 4.61) | 2.12 (1.18, 3.53) | −0.321 | −0.139 |
| Basal AFC | 8.0 (7.0, 11.0) | 8.0 (5.0, 12.0) | −0.001 | 8.0 (7.0, 11.0) | 5.0 (3.0, 10.0) | −0.400 | 0.033 |
| Infertility duration (years) | 2.0 (1.5, 4.0) | 3.0 (1.5, 5.0) | 0.131 | 2.0 (1.5, 4.0) | 3.0 (2.0, 4.0) | 0.032 | 0.061 |
| Primary infertility, n (%) | 29 (64.4) | 119 (53.8) | −0.215 | 27 (65.9) | 24 (58.5) | −0.149 | 0.009 |
| Bilateral OMA, n (%) | 13 (28.9) | 85 (38.5) | 0.202 | 13 (31.7) | 13 (31.7) | 0.000 | 0.047 |

**Notes**: Propensity score matching (PSM) was performed to reduce selection bias between the non-surgical (Group B) and surgical (Group C) management groups. The propensity score was estimated using a logistic regression model that included the following covariates: age, BMI, AMH, AFC, infertility duration, primary infertility status, and bilateral OMA status. One-to-one nearest-neighbor matching without replacement was performed using a caliper width of 0.2 standard deviations of the logit of the propensity score. The standardized mean difference (SMD) was used to assess covariate balance before and after matching; an SMD <0.1 is generally considered to indicate adequate balance. Inverse probability of treatment weighting (IPTW) was performed as a sensitivity analysis using stabilized weights to reduce the influence of extreme weights. Data are presented as median (Q1, Q3) for continuous variables and n (%) for categorical variables.

**Abbreviations**: AFC, antral follicle count; AMH, anti-Müllerian hormone; BMI, body mass index; IPTW, inverse probability of treatment weighting; OMA, ovarian endometrioma; PSM, propensity score matching; Q1, first quartile; Q3, third quartile; SMD, standardized mean difference.

**Table S8. Multivariable Logistic Regression: Independent Predictors of Cumulative Live Birth Rate (Group A vs Group B)**

| **Variable** | **β** | **OR (95% CI)** | **P value** |
| --- | --- | --- | --- |
| Group (reference: Group A) | | |  |
| Group B | 0.06 | 1.06 (0.50-2.22) | 0.882 |
| Age | -0.03 | 0.97 (0.87-1.08) | 0.537 |
| BMI | 0.07 | 1.08 (0.96-1.20) | 0.199 |
| AMH | -0.07 | 0.93 (0.83-1.04) | 0.199 |
| Retrieved oocytes | 0.07 | 1.07 (0.99-1.15) | 0.096 |
| Top-quality embryos | 0.04 | 1.04 (0.87-1.24) | 0.670 |
| First transfer cycle type  (reference: fresh cycle) | | | |
| Frozen-thawed cycle | -0.10 | 0.91 (0.40-2.05) | 0.817 |
| Number of transferred embryos  (reference: 1) | | | |
| 2 embryos | 0.74 | 2.10 (0.82-5.36) | 0.122 |
| Type of transferred embryos  (reference: cleavage-stage) | | | |
| Blastocyst | 0.11 | 1.12 (0.36-3.44) | 0.846 |
| Endometrial thickness at transfer | -0.00 | 1.00 (0.87-1.13) | 0.949 |

**Notes**: Multivariable logistic regression analysis with cumulative live birth as the dependent variable. The model included variables with P < 0.10 in univariable analysis and clinically relevant confounders (age, BMI, AMH). The Hosmer-Lemeshow test was used to assess goodness-of-fit (P > 0.05 indicates adequate fit). The area under the receiver operating characteristic curve (AUC-ROC) is reported to assess discriminative ability. Nagelkerke R² indicates the proportion of variance explained by the model.

**Abbreviations**: AFC, antral follicle count; AMH, anti-Müllerian hormone; AUC, area under the curve; BMI, body mass index; CI, confidence interval; OMA, ovarian endometrioma; OR, odds ratio; ROC, receiver operating characteristic.

**Table S9. Exploratory Subgroup Analyses: Effect of Surgical Status on Cumulative Live Birth Rate (Group B vs. Group C)**

| **Subgroup** | **Group B** |  | **Group C** |  | **OR (95% CI)** | **P** | **P-interaction** |
| --- | --- | --- | --- | --- | --- | --- | --- |
|  | n/N | Rate (%) | n/N | Rate (%) |  |  |  |
| Overall | 28/47 | 59.6 | 121/231 | 52.4 | 0.75 (0.39–1.41) | 0.368 | — |
| Age |  |  |  |  |  |  |  |
| <35 years | 26/41 | 63.4 | 102/183 | 55.7 | 0.73 (0.36–1.46) | 0.370 |  |
| ≥35 years | 2/6 | 33.3 | 19/48 | 39.6 | 1.31 (0.22–7.76) | 0.766 | 0.548 |
| AMH level |  |  |  |  |  |  |  |
| ≥1.1 ng/mL (Normal) | 25/41 | 61.0 | 94/173 | 54.3 | 0.76 (0.38–1.53) | 0.442 |  |
| <1.1 ng/mL (Low) | 1/4 | 25.0 | 23/49 | 46.9 | 2.65 (0.26–27.4) | 0.406 | 0.885 |
| Embryo type at transfer |  |  |  |  |  |  |  |
| Cleavage-stage | 18/30 | 60.0 | 100/186 | 53.8 | 0.78 (0.35–1.70) | 0.525 |  |
| Blastocyst | 10/17 | 58.8 | 21/45 | 46.7 | 0.61 (0.20–1.90) | 0.395 | 0.737 |
| OMA laterality |  |  |  |  |  |  |  |
| Unilateral | 20/34 | 58.8 | 74/143 | 51.7 | 0.75 (0.35–1.60) | 0.458 |  |
| Bilateral | 8/13 | 61.5 | 47/88 | 53.4 | 0.72 (0.22–2.36) | 0.584 | 0.948 |

**Notes**: Exploratory subgroup analyses were performed to assess whether the effect of surgical status (Group B vs. Group C) on cumulative live birth rate varied across clinically relevant patient subgroups. Subgroups were defined a priori based on: (1) age (<35 vs. ≥35 years, based on established fertility decline thresholds); (2) ovarian reserve (AMH ≥1.1 vs. <1.1 ng/mL, based on the Bologna criteria for poor ovarian response); (3) embryo type at transfer (cleavage-stage vs. blastocyst); and (4) OMA laterality (unilateral vs. bilateral). Odds ratios (OR) were calculated using logistic regression with Group B (non-surgical) as the reference category; therefore, OR <1 indicates higher odds of live birth in Group C (surgical). The P-interaction was derived from a likelihood ratio test comparing models with and without the interaction term (subgroup × treatment group), testing whether the treatment effect differs significantly between subgroups. A P-interaction <0.05 would suggest significant effect modification by the subgroup factor. Given the exploratory nature of these analyses and the limited sample sizes in some subgroups, results should be interpreted with caution and considered hypothesis-generating.

**Abbreviations**: AMH, anti-Müllerian hormone; CI, confidence interval; OMA, ovarian endometrioma; OR, odds ratio; P-int, P-value for interaction.

**Table S10. Sensitivity Analysis: Effect of Adding Bilateral OMA Status as Covariate**

| **Comparison** | **Outcome** | **N** | **Original Model** |  | **Adjusted Model (+Bilateral)** | | **Change (%)** | **Bilateral Effect** |
| --- | --- | --- | --- | --- | --- | --- | --- | --- |
|  |  |  | Effect (95% CI) | P | Effect (95% CI) | P |  |  |
| A vs. B | Cumulative LBR | 157 | OR = 1.07 (0.52–2.21) | 0.861 | OR = 1.08 (0.52–2.24) | 0.843 | +0.9 | OR = 0.63 (0.29–1.35)  P = 0.231 |
| A vs. B | Retrieved oocytes | 157 | β = 0.14 (−1.82, 2.10) | 0.887 | β = 0.13 (−1.82, 2.08) | 0.893 | −5.6 | β = 1.65 (−0.44, 3.74)  P = 0.122 |
| B vs. C | Cumulative LBR | 266 | OR = 0.77 (0.39–1.52) | 0.453 | OR = 0.77 (0.39–1.53) | 0.459 | +0.3 | OR = 0.97 (0.58–1.62)  P = 0.918 |
| B vs. C | Retrieved oocytes | 266 | β = −2.28 (−3.77, −0.80) | 0.003** | β = −2.34 (−3.83, −0.86) | 0.002** | −2.6 | β = 0.66 (−0.49, 1.82)  P = 0.259 |

**Notes**: Sensitivity analysis was conducted to evaluate whether bilateral OMA status (vs. unilateral) confounded the association between group allocation and outcomes. Bilateral OMA was added as a covariate to the primary multivariable regression models, and the change in effect estimates was assessed. A change of >10% in the effect estimate would indicate meaningful confounding by bilateral OMA status, potentially warranting its inclusion as a covariate in all analyses. For cumulative live birth rate (binary outcome), logistic regression was used with the OR as the effect estimate. For retrieved oocyte number (continuous outcome), linear regression was used with the β coefficient (mean difference) as the effect estimate. The "Bilateral Effect" column shows the independent effect of bilateral OMA status (vs. unilateral) on each outcome within the adjusted models. All changes in effect estimates were <10%, confirming that bilateral OMA status did not meaningfully confound the primary findings and supporting the robustness of the unadjusted analyses.

Statistical significance: **P < 0.01.

**Abbreviations**: CI, confidence interval; LBR, live birth rate; OMA, ovarian endometrioma; OR, odds ratio.

**Table S11. Missing data summary for key variables across study groups**

| **Variable** | **Group A (n = 116)** | **Group B (n = 47)** | **Group C (n = 231)** |
| --- | --- | --- | --- |
|  | **Available / Missing (%)** | **Available / Missing (%)** | **Available / Missing (%)** |
| ***Demographics*** | | | |
| Age | 116 / 0 (0.0%) | 47 / 0 (0.0%) | 231 / 0 (0.0%) |
| BMI | 116 / 0 (0.0%) | 47 / 0 (0.0%) | 231 / 0 (0.0%) |
| Infertility duration | 116 / 0 (0.0%) | 47 / 0 (0.0%) | 231 / 0 (0.0%) |
| Infertility type | 116 / 0 (0.0%) | 47 / 0 (0.0%) | 231 / 0 (0.0%) |
| ***Ovarian reserve markers*** | | | |
| AMH | 112 / 4 (3.4%) | 45 / 2 (4.3%) | 223 / 8 (3.5%) |
| AFC | 116 / 0 (0.0%) | 47 / 0 (0.0%) | 228 / 3 (1.3%) |
| Basal FSH | 108 / 8 (6.9%) | 42 / 5 (10.6%) | 217 / 14 (6.1%) |
| Basal LH | 108 / 8 (6.9%) | 42 / 5 (10.6%) | 218 / 13 (5.6%) |
| Basal E2 | 108 / 8 (6.9%) | 42 / 5 (10.6%) | 217 / 14 (6.1%) |
| Basal P | 95 / 21 (18.1%) | 36 / 11 (23.4%) | 198 / 33 (14.3%) |
| Basal T | 93 / 23 (19.8%) | 36 / 11 (23.4%) | 198 / 33 (14.3%) |
| ***COS parameters*** | | | |
| Gn total dose | 116 / 0 (0.0%) | 47 / 0 (0.0%) | 230 / 1 (0.4%) |
| Gn duration | 116 / 0 (0.0%) | 47 / 0 (0.0%) | 230 / 1 (0.4%) |
| E2 on trigger day | 106 / 10 (8.6%) | 44 / 3 (6.4%) | 213 / 18 (7.8%) |
| Endometrial thickness | 115 / 1 (0.9%) | 47 / 0 (0.0%) | 229 / 2 (0.9%) |
| ***Oocyte and embryo outcomes*** | | | |
| Retrieved oocytes | 116 / 0 (0.0%) | 47 / 0 (0.0%) | 229 / 2 (0.9%) |
| MII oocytes | 116 / 0 (0.0%) | 47 / 0 (0.0%) | 231 / 0 (0.0%) |

**Notes:** Data are presented as number of patients with available data / number of patients with missing data (percentage missing). Group A: non-surgical management with OMA <4 cm; Group B: non-surgical management with OMA ≥4 cm; Group C: surgical management (laparoscopic cystectomy) with OMA ≥4 cm. Missing data for AMH occurred primarily in patients treated during 2016–2017, when AMH testing was not yet routinely performed prior to IVF cycles. Missing AFC data in Groups B and C resulted from incomplete documentation of bilateral antral follicle counts in ultrasound reports. Missing basal hormone data (FSH, LH, E2, P, T) occurred when cycle day 2–3 blood sampling was not performed or values were not recorded in medical records. The proportion of missing data for covariates used in multivariable analyses (age, BMI, AMH, AFC, infertility duration, infertility type) was <5% in all groups. Complete case analysis was used for all statistical analyses.

**Abbreviations:** AFC, antral follicle count; AMH, anti-Müllerian hormone; BMI, body mass index; COS, controlled ovarian stimulation; E2, estradiol; FSH, follicle-stimulating hormone; Gn, gonadotropin; IVF, in vitro fertilization; LH, luteinizing hormone; MII, metaphase II; OMA, ovarian endometrioma; P, progesterone; T, testosterone.

**Table S12. E-value analysis for unmeasured confounding in cumulative live birth rate comparisons**

| **Comparison** | **Analytical method** | **OR** | **95% CI** | **P value** | **E-value (point estimate)** | **E-value (CI limit)** |
| --- | --- | --- | --- | --- | --- | --- |
| Group A vs B | Multivariable logistic regression | 1.06 | 0.50–2.22 | 0.882 | 1.31 | 1.00 |
| Group B vs C | Multivariable logistic regression | 0.77 | 0.38–1.56 | 0.469 | 1.92 | 1.00 |
| Group B vs C | Propensity score matching | 0.80 | 0.37–1.73 | 0.571 | 1.81 | 1.00 |
| Group B vs C | Inverse probability of treatment weighting | 0.71 | 0.35–1.44 | 0.343 | 2.17 | 1.00 |

**Notes:** E-values were calculated to assess the potential impact of unmeasured confounding on the observed associations. The E-value for the point estimate represents the minimum strength of association (on the risk ratio scale) that an unmeasured confounder would need to have with both the exposure and outcome, conditional on measured covariates, to fully explain away the observed association. The E-value for the confidence interval (CI) limit represents the minimum confounding strength needed to shift the CI to include the null. An E-value of 1.00 for the CI limit indicates that the 95% CI already crosses the null value of 1.0, meaning that any degree of unmeasured confounding could potentially explain the observed association. For protective associations (OR < 1), E-values were calculated using the inverse of the OR.

**Abbreviations:** CI, confidence interval; CLBR, cumulative live birth rate; E-value, expected value for unmeasured confounding; IVF, in vitro fertilization; OMA, ovarian endometrioma; OR, odds ratio.
